# Quality control in microarray assessment of gene expression in human airway epithelium

**DOI:** 10.1186/1471-2164-10-493

**Published:** 2009-10-24

**Authors:** Tina Raman, Timothy P O'Connor, Neil R Hackett, Wei Wang, Ben-Gary Harvey, Marc A Attiyeh, David T Dang, Matthew Teater, Ronald G Crystal

**Affiliations:** 1 Department of Genetic Medicine, Weill Cornell Medical College, New York, New York, USA; 2 DNA Microarray Core, Life Sciences Core Laboratories Center, Cornell University, Ithaca, New York, USA; 3 Division of Pulmonary and Critical Care Medicine, Weill Cornell Medical College, New York, New York, USA

## Abstract

**Background:**

Microarray technology provides a powerful tool for defining gene expression profiles of airway epithelium that lend insight into the pathogenesis of human airway disorders. The focus of this study was to establish rigorous quality control parameters to ensure that microarray assessment of the airway epithelium is not confounded by experimental artifact. Samples (total n = 223) of trachea, large and small airway epithelium were collected by fiberoptic bronchoscopy of 144 individuals and hybridized to Affymetrix microarrays. The pre- and post-chip quality control (QC) criteria established, included: (1) RNA quality, assessed by RNA Integrity Number (RIN) ≥ 7.0; (2) cRNA transcript integrity, assessed by signal intensity ratio of GAPDH 3' to 5' probe sets ≤ 3.0; and (3) the multi-chip normalization scaling factor ≤ 10.0.

**Results:**

Of the 223 samples, all three criteria were assessed in 191; of these 184 (96.3%) passed all three criteria. For the remaining 32 samples, the RIN was not available, and only the other two criteria were used; of these 29 (90.6%) passed these two criteria. Correlation coefficients for pairwise comparisons of expression levels for 100 maintenance genes in which at least one array failed the QC criteria (average Pearson r = 0.90 ± 0.04) were significantly lower (p < 0.0001) than correlation coefficients for pairwise comparisons between arrays that passed the QC criteria (average Pearson r = 0.97 ± 0.01). Inter-array variability was significantly decreased (p < 0.0001) among samples passing the QC criteria compared with samples failing the QC criteria.

**Conclusion:**

Based on the aberrant maintenance gene data generated from samples failing the established QC criteria, we propose that the QC criteria outlined in this study can accurately distinguish high quality from low quality data, and can be used to delete poor quality microarray samples before proceeding to higher-order biological analyses and interpretation.

## Background

The assessment of gene expression of the human transcriptome using microarray technology is a powerful tool for identifying genes and gene expression patterns involved in mechanisms of normal organ function and the pathogenesis of disease [1-3]. Microarray technology is ideal for studies of the human airway epithelium in health and disease in that the airway is one of the few internal organs where it is possible to repetitively sample sufficient quantities of pure populations of parenchymal cells from healthy individuals as well as individuals with lung disease [4-11]. In this regard, we and several other groups have used human gene expression microarrays to assess the expression of genes in the human airway epithelium, cell populations easily attainable via fiberoptic bronchoscopy [4,9,12-15].

While it is easy to obtain the cells, the output from microarray data critically depends on the quality of the RNA and the cRNA derivatives hybridized to the microarray [16-27]. Although several different cutoff criteria for RNA integrity and microarray data quality have been proposed, they are not consistently applied. In this context, the focus of this study is to establish rigorous quality control (QC) criteria to ensure high quality data from arrays that is comparable and reproducible among different investigators and laboratories. Our strategy is based on the concept that the quality of expression data can be efficiently assessed using three discreet QC metrics computed on the sample and chip level, and that application of these metrics can ensure uniformly high quality microarray data. Using Affymetrix Human Genome U133 Plus 2.0 arrays to sample a total of 223 samples of tracheal, and large and small airway epithelium from 144 individuals [healthy non-smokers, healthy smokers, symptomatic smokers, smokers with lone emphysema with normal spirometry, and smokers with COPD (GOLD I - III)], we have established pre- and post-chip QC criteria based on empirical observations of our data in conjunction with published suggestions that include: (1) RNA quality, assessed by RNA Integrity Number; (2) cRNA transcript integrity, assessed by signal intensity ratio of the glyceraldehyde-3-phosphate dehydrogenase (GAPDH) 3' to 5' probe sets; and (3) a defined upper limit for the multi-chip normalization scaling factor. Of the 223 samples, all three criteria were assessed in 191; of these 184 (96.3%) passed all three criteria. For the remaining 32 samples, the RIN was not available, and only the other two criteria were used; of these 29 (90.6%) passed these two criteria. Expression data for 100 maintenance gene probe sets on the array demonstrates that among the samples failing QC criteria, there is greater variability among reported expression levels for maintenance genes compared to randomly selected samples passing the QC criteria. The QC criteria proposed in this study should provide a useful guideline for future studies using microarrays to assess mRNA levels in human airway epithelial samples, and should be adaptable to assessment of microarray data from other cell populations.

Some of the results of these studies have been previously reported in the form of an abstract [28].

## Results

### Airway Epithelium

A total of 223 samples of airway epithelium were obtained by bronchial brushing from three different locations (trachea, large airway, small airway) from 144 subjects with 5 different pulmonary phenotypes (healthy non-smokers, healthy smokers, symptomatic smokers, smokers with lone emphysema with normal spirometry, and smokers with COPD; Table 1). The mean ages varied from 36 to 52 yr, and males represented the majority in all but one group. The ancestries varied among those of European, Hispanic, Asian and African. The lung function fit the criteria for each group. A range of 4.4 to 7.6 × 10^6 ^cells were recovered from trachea, large airway and small airway in all five pulmonary phenotypic groups and cell counts were not dependent upon phenotype of the subject or site of bronchial brushing (p > 0.05 by ANOVA). From all locations, an average of 99 to 100% of all cells recovered were epithelial with less than 1% contamination by non-epithelial cells. The cell differentials varied depending on location as previously described [4,29,30]. The average yield of extracted RNA was 25.3 ± 10 μg. varying from 3.5 to 53.9 μg.

**Table 1:** Demographic of the Study Population and Biologic Samples^1^

**Parameter**	**Healthy nonsmoker**			**Healthy smoker**			**Symptomatic smoker**			**Lone emphysema with normal spirometry**^**6**^	**COPD**^**7**^
			
	**Trachea**	**Large airway**	**Small airway**	**Trachea**	**Large airway**	**Small airway**	**Trachea**	**Large airway**	**Small airway**	**Small airway**	**Small airway**
				
n	17	21	35	15	32	44	3	4	10	22	20
Age	42 ± 7	42 ± 9	43 ± 10	43 ± 7	44 ± 6	44 ± 6	36 ± 6	39 ± 6	41 ± 10	49 ± 7	52 ± 8
Gender^2^	13/4	15/6	26/9	13/2	22/10	31/13	2/1	3/1	5/5	16/6	16/4
Ancestry^3^	6/4/0/7	8/3/1/9	15/4/1/15	5/5/0/5	7/4/0/21	11/4/0/29	0/2/0/1	1/2/0/1	3/3/0/4	4/1/0/17	8/4/1/7
Smoking history	<1.0	<1.0	<1.0	28 ± 16	28 ± 18	28 ± 16	14 ± 4	16 ± 9	21 ± 13	31 ± 18	38 ± 23
Pulmonary function parameters^4^											
FVC	111 ± 16	105 ± 11	109 ± 11	108 ± 11	109 ± 12	109 ± 12	116 ± 6	113 ± 7	110 ± 109	102 ± 11	93 ± 23
FEV1	111 ± 18	101 ± 26	105 ± 21	108 ± 13	109 ± 13	109 ± 14	112 ± 16	112 ± 13	108 ± 20	97 ± 12	72 ± 22
FEV1/FVC	83 ± 7	82 ± 6	80 ± 7	82 ± 6	81 ± 5	81 ± 5	80 ± 6	81 ± 3	81 ± 13	79 ± 4	61 ± 9
TLC	106 ± 17	99 ± 14	104 ± 13	100 ± 8	102 ± 12	100 ± 12	106 ± 4	108 ± 4	104 ± 19	93 ± 13	105 ± 22
DLCO	110 ± 9	101 ± 18	101 ± 17	94 ± 7	96 ± 11	96 ± 11	92 ± 14	95 ± 13	94 ± 18	65 ± 8	73 ± 19
Average # of cells recovered (×10^6^)	5.4	6.8	5.7	4.4	6.4	6.4	6.6	7.6	6.2	5.9	6.3
Cell differential^5^											
% epithelial	100 ± 0.2	100 ± 0.7	100 ± 0.6	100 ± 0.2	100 ± 0.7	100 ± 0.5	100 ± 0.0	100 ± 0.6	100 ± 0.4	99 ± 0.8	99 ± 1.7
% inflammatory	0.1 ± 0.2	0.3 ± 0.7	0.3 ± 0.6	0.1 ± 0.2	0.3 ± 0.7	0.2 ± 0.5	0.0	0.4 ± 0.6	0.4 ± 0.4	0.6 ± 0.8	1.5 ± 1.7
% ciliated	49 ± 7.1	55 ± 3.9	77 ± 5.6	27 ± 8.2	49 ± 9.0	72 ± 6.7	26 ± 2.8	47 ± 16	76 ± 5.4	73 ± 8.7	69 ± 2.8
% secretory	6.6 ± 4.0	12 ± 4.0	6.8 ± 3.5	8.8 ± 4.4	11 ± 4.1	7.1 ± 3.0	12 ± 4.6	14 ± 1.2	5.9 ± 3.0	9.7 ± 7.2	12 ± 2.9
% basal	29 ± 8.6	20 ± 3.4	9.1 ± 3.4	39 ± 5.2	24 ± 5.6	9.9 ± 3.3	37 ± 5.4	17 ± 10	10 ± 2.5	9.9 ± 4.8	8.2 ± 2.3
% undifferentiated	15 ± 6.0	13 ± 3.8	7.3 ± 3.6	25 ± 11	15 ± 7.7	11 ± 5.6	25 ± 1.0	22 ± 6.0	7.8 ± 1.1	7.3 ± 2.4	9.6 ± 1.8

^1 ^Data is presented as mean ± standard deviation.

^2 ^Male/Female.

^3 ^European/Hispanic/Asian/African.

^4 ^Pulmonary function testing parameters are given as percent of predicted value with the exception of FEV1/FVC, which is reported as % observed; FVC - forced vital capacity, FEV1 - forced expiratory volume in 1 sec, TLC - total lung capacity, DLCO - diffusing capacity. For individuals with COPD, FVC, FEV1, and FEV1/FVC are post-bronchodilator values.

^5 ^% epithelial and inflammatory are based on the total number of cells recovered; % ciliated, secretory, basal and undifferentiated cells are based on the total number of epithelial cells recovered.

^6 ^Lone emphysema with normal spirometry smokers.

^7 ^COPD smoker, GOLD stage I n = 9, II n = 9, III n = 2.

### Establishment and Testing of Quality Control Criteria

The overall strategy was to utilize the data on 223 samples to establish prospectively applicable QC criteria that would ensure high quality expression microarray data for biological interpretation in our ongoing studies. The QC criteria were selected as rigorous and objective quality control metrics at three distinct stages of the microarray workflow, and were applied to all 223 samples hybridized to microarray in this study; for the RIN assessment, the n = 191 (32 samples were unavailable for RIN analysis because the samples were hybridized to microarray prior to the development of the Bioanalyzer RIN software). For the GAPDH 3'/5' signal intensity ratio and scaling factor criteria, all 223 samples were included.

Of the 223 samples, all three criteria were assessed in 191; of these 184 (96.3%) passed all three criteria. For the remaining 32 samples, the RIN was not available, and only the other two criteria were used; of these 29 (90.6%) passed these two criteria. Only 10 (4.5%) failed at least one QC criterion, and were therefore considered to have failed QC. The overall breakdown of samples failing QC was: 2 large airway samples (1 healthy non-smoker and 1 healthy smoker) and 8 small airway samples (1 healthy smoker, 4 symptomatic smokers, and 3 smokers with COPD). The greatest source of failure was the scaling factor criterion, which contributed to 70% of the overall failures. All of the 10 samples failing the QC criteria failed the RIN and/or scaling factor criterion, indicating that these metrics may be the most sensitive to technical variance, and therefore are central to assessing overall array quality. While 7 samples failed by one criterion each, 1 sample failed by both the RIN and GAPDH 3'/5' ratio criteria, and 2 samples failed by both the RIN and scaling factor criteria, suggesting that the quality control parameters exert correlated effects on array performance.

### RIN

**Figure 1:**
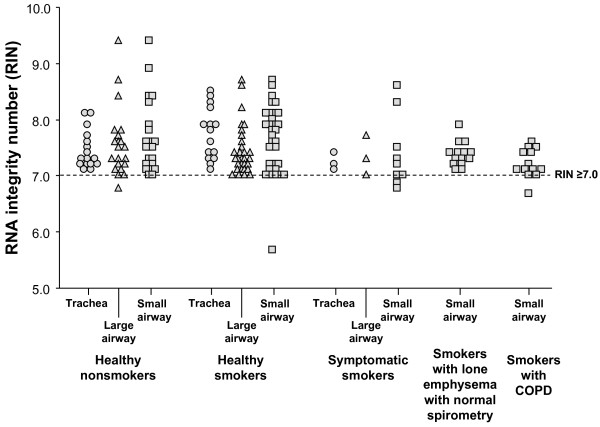
**Assessment of RNA quality in airway epithelial samples**. Integrity of 180 RNA samples was scored using the RNA Integrity Number (RIN) generated by Agilent 2100 Bioanalyzer Software (1 = highly degraded; 10 = intact). Samples are grouped by phenotype as defined in Methods, and within each phenotype the site of the epithelial sample is indicated (trachea; large airway; small airway). Samples with RIN ≥ 7.0, shown by the dotted line, passed QC criterion, while the 5 samples below the dotted line failed the QC criterion.

The RNA quality was examined by the Bioanalyzer-generated RIN score in 191 samples for which there was data available (see above). Based on published data [26,31-33], samples with a RIN ≤ 7.0 were designated to have passed QC (Figure 1). Five out of the 191 samples (2.6%) had RIN scores <7.0. The RIN values were not significantly dependent upon the phenotype or biologic origin of the RNA sample (p > 0.1 by ANOVA), with n = 4 small airway samples (1 healthy smoker, 2 symptomatic smokers, 1 smoker with COPD) and 1 large airway sample (healthy nonsmoker) failing on the basis of RIN <7.0.

### GAPDH 3'/5' Signal Intensity Ratio

**Figure 2:**
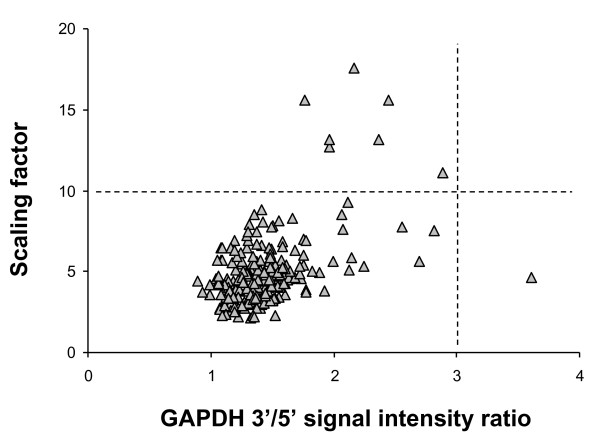
**Assessment of GAPDH 3'/5' and Chip scaling factor**. Ratios of signal intensities for GAPDH 3' and 5' probe sets for 223 samples were extracted from the GeneChip Operating Software (GCOS) Quality Report and plotted against the Scaling Factors analyzed with a target intensity value of 500 extracted from the GCOS Quality Report. Samples with GAPDH 3'/5' ratio ≤ 3.0, to the left of the vertical dotted line, passed QC criterion, while the one sample to the right of the dotted line failed the QC criterion. Samples with scaling factor values ≤ 10.0 passed QC criterion (below the horizontal dashed line) while the 7 samples above the dashed line failed the QC criterion.

As a metric for the efficiency of transcription and amplification of antisense cRNA from the cDNA derivative of the starting RNA material, the signal intensities for the probe sets for GAPDH residing at the 5' end and within the 600 nucleotides most proximal to the priming site at the 3' end of the transcript were compared. For all samples hybridized to microarrays, 3' to 5' probe set intensities for the GAPDH gene were extracted to compute the 3'/5' signal intensity ratio. Based on published data [16,23,34-36], the criterion for passing QC was established as GAPDH 3'/5' ratio ≤ 3.0 (Figure 2). By this criterion, only 1 small airway sample from a symptomatic smoker failed QC. The Affymetrix expression microarray also returns 3'/5' ratios for other genes including β-actin. But due to the strong correlation in 3'/5' ratios for β-actin and GAPDH (r^2 ^= 0.92; p < 0.0001), application of addition cutoff criteria beyond GAPDH was considered redundant. In the context of airway epithelium, although GAPDH is not an ideal "housekeeping" gene as its expression may vary under different conditions, this does not interfere with its use in assessing cRNA quality [37].

### Multi-chip Normalization Scaling Factor

The scaling factor was used as an overall index of the microarray hybridization, washing, and scanning process. Scaling factor values for all 223 samples computed at a target intensity value of 500 were examined. The criterion of scaling factor values ≤ 10.0 was established (Figure 2). Seven out of the 223 samples (3.1%) had scaling factor values above the acceptable cutoff. The scaling factor values were not significantly dependent upon the phenotype or biologic origin of the sample (p > 0.1 by ANOVA), with n = 5 small airway samples (1 healthy smoker, 2 symptomatic smokers, 2 smokers with COPD) and n = 2 large airway samples (1 healthy nonsmoker, 1 healthy smoker) failing on the basis of scaling factor >10.0.

The interdependence of failing different QC criteria was assessed (Table 2). Of the total 7 samples that failed RIN, three failed one of other the other QC criteria with 1 failing GAPDH 3'/5' test and 2 failing scaling factor test. There was no pattern of repeated QC failure by a single subject sampled on more than one occasion, neither was there correlation of failure with differential or % non-epithelial contamination.

**Table 2:** Classification of Quality Control Failures by Criterion ^1^

	**RIN2**	**GAPDH 5'/3'**	**Scaling factor**
**Alone**	2	0	5
**+ RIN**	0	1	2
**+ GAPDH 5'/3'**	1	0	0
**+ Scaling factor**	2	0	0

**Total**	5	1	7

^1 ^The 233 samples were assessed by the established QC criteria and all those failing one or more were classified by which QC criteria were failed.

^2 ^Only 191 of the 223 samples were assessed for RIN.

### Maintenance Gene Expression Levels

**Figure 3:**
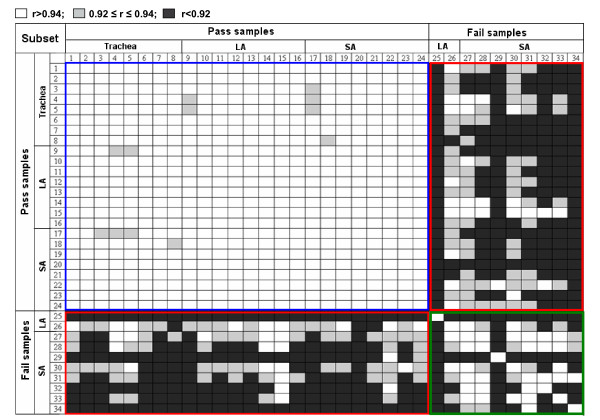
**Pairwise correlations of expression levels for 100 maintenance genes**. Expression levels for 100 maintenance genes were determined for 34 airway epithelial samples of which 24 randomly selected samples passed the pre-determined QC criteria and 10 failed one or more of the criteria. The vertical and horizontal numbers refer to the 34 samples, categorized as "pass" or "fail"; LA = large airway; SA = small airway. Pearson correlation coefficients for all pairwise comparisons between the 34 samples were determined and are plotted in grey-scale, with each cell representing a single correlation between two samples (white, r > 0.94; gray, 0.92 ≤ r ≤ 0.94; black, r < 0.92). Shown are the 24 × 24 comparison of samples both passing QC, the 24 × 10 between samples passing QC and samples failing QC, and the 10 × 10 comparison of samples both failing QC. Note that all of the correlation values <0.92 are derived only from pairwise comparisons including samples failing the QC criteria.

**Figure 4:**
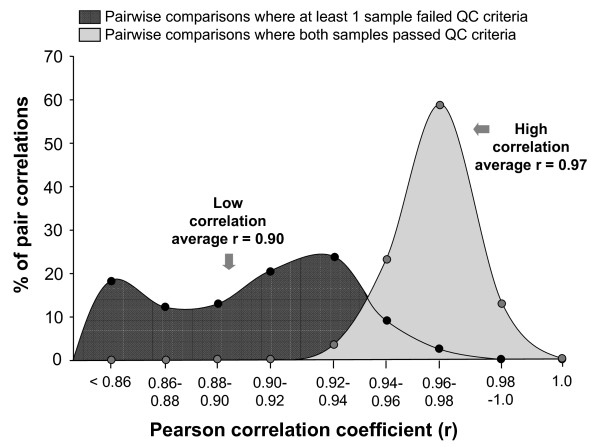
**Frequency distribution of correlation coefficients calculated for pairwise comparisons**. Shaded dark grey region represents pairwise comparisons (n = 285) where at least 1 sample failed the QC criteria. Light grey region represent pairwise comparisons (n = 276) where both samples pass QC criteria. The majority of samples passing the QC criteria have correlation values >0.94.

To assess whether the gene expression data derived from samples that pass all of the QC criteria was more robust than that derived from samples that failed one or more conditions, for every sample, regardless of QC metric values, expression levels were extracted for the 100 maintenance genes. For the 10 samples failing QC criteria and 24 randomly selected samples passing the QC criteria, the expression profile for all 100 genes was compared. Pearson's correlation was calculated for all pairwise comparisons (i.e., 24 × 24 comparison of samples both passing QC, 24 × 10 among samples passing QC and samples failing QC, and 10 × 10 comparison of samples both failing QC). Correlation coefficient values indicated that samples passing QC criteria were highly correlated with other samples passing QC criteria (average Pearson r = 0.97) while samples failing QC criteria showed lower correlations with all other samples (average Pearson r = 0.90; Figure 3). The range of correlation coefficient values obtained for pairwise correlations of samples passing QC criteria was 0.92 to 0.99. In contrast, when comparing samples failing QC criteria with all other samples, the range of correlation coefficient values was 0.76 to 0.97. There was no difference in the correlation coefficient values for samples failing QC for RIN criterion versus other causes (p > 0.4). The distribution of correlation coefficients for the pairwise comparisons of samples passing QC criteria was significantly different from the distribution of values for pairwise comparisons where at least one sample failed the QC criteria (p < 0.0001, Mann-Whitney *U *Test; Figure 4).

**Figure 5:**
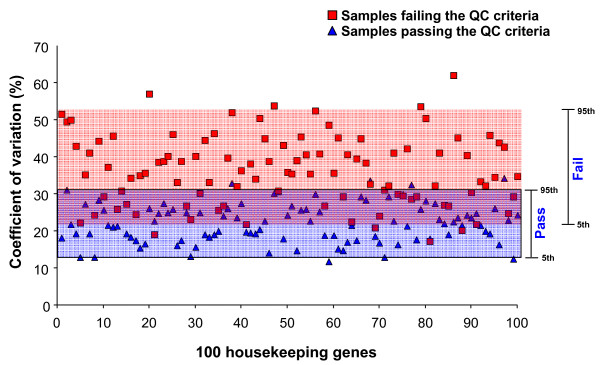
**Variability in maintenance gene expression levels in samples that pass or fail QC criteria**. The coefficients of variation for each of the 100 maintenance genes were calculated across 2 data sets: a data set of 10 samples failing QC criteria (red squares), and a randomly selected data set of 10 samples that pass QC criteria (blue triangles). Upper and lower boundaries of shaded regions represent 95^th ^and 5^th ^percentiles, respectively, of coefficient of variation across samples failing the QC criteria (red box) and coefficient of variation across samples passing the QC criteria (blue box).

Of the 24 samples passing QC criteria that were used for the correlation matrix analysis, 10 samples matched in airway location with the 10 samples failing QC criteria were selected to assess coefficient of variation of each of the 100 maintenance genes. Expression levels for the 100 maintenance genes showed significantly greater variability among the 10 samples failing QC criteria ("fail" data set) than among the 10 samples passing QC criteria ("pass" data set, Figure 5). Across the "pass" data set, the median coefficient of variation for the maintenance genes was 21.7% (5^th ^to 95^th ^percentile 13.0 to 31.0%). By contrast, across the "fail" data set, the median coefficient of variation for the 100 genes was 35.7% (5^th ^to 95^th ^percentile 21.8 to 52.5%; p < 0.0001, Mann-Whitney *U *test).

Similarly, the coefficient of variation for all probe sets was greater for microarrays that failed QC compared to that for microarrays that passed. Two datasets of 9 microarrays each were compared giving a mean coefficient of variation of 34 ± 0.1% for the arrays that passed QC and 43 ± 0.1% for the arrays that failed QC. The impact on discovery of biological differences (for example impact of smoking on gene expression profile [12]), was assessed by power calculations. If two groups of 15 smokers and 15 non-smokers were compared, the required true difference of means for detection with p < 0.05 with and power of 0.95 rises from 0.46 with arrays that pass QC to 0.58 with arrays that failed QC (i.e., small biological effects become more difficult to detect).

To examine potential causes of the variation in maintenance gene expression levels unrelated to the QC criteria, differences among the subjects were assessed. The 223 airway epithelial samples acquired for this study were derived from 144 individuals, as it was possible for a single individual to undergo bronchial brushing at one or more of the three target sites: trachea, large airway, and small airway. By independent linear regression, there was no correlation of gene expression level for the 100 maintenance genes (r^2^<0.05 for all genes) with age (average 45 ± 8.8) across the 144 individuals from whom airway epithelium was derived. None of the genes showed strong correlation (r^2^<0.15) with smoking history (average pack-yr 30 ± 18). Correlation analysis of expression levels with pulmonary function parameters showed no relationship (r^2^<0.09 for all genes with all parameters).

### Impact of QC Failures on Global Lung Biology

**Figure 6:**
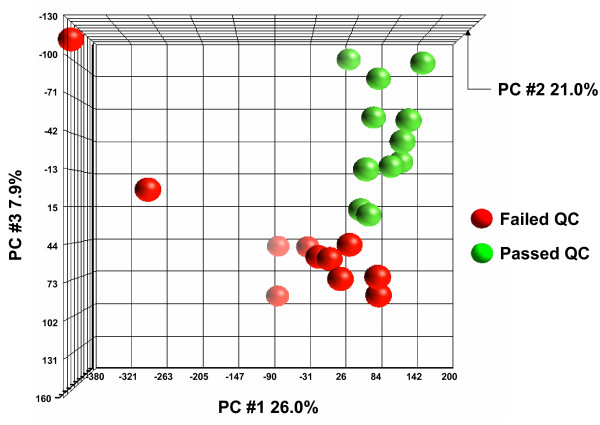
**Principal components analysis of genome-wide gene transcriptome data in failed and passed COPD subjects**. The axes have been rotated presenting a top view to highlight the 2 standard deviation ovoid clustering of expression from failed and passed COPD subjects. Each axis represents one principal component (PC), with PC1 on the x axis, PC3 on the y axis and PC2 on the z axis. Failed COPD subjects are represented by red spheres and passed COPD subjects by green spheres.

In order to assess the functional consequences of the QC criteria on the gene expression data, a Principal Components Analysis (PCA) was used to compared samples that passed QC to those that failed. For this analysis, an independent set of microarray data that failed QC was available from a technician training program in the Weill Cornell Medical College Department of Genetic Medicine. From this training program, 11 microarrays that failed QC were available from small airway epithelium samples collected from individuals with COPD (n = 1 failed due to the RIN criteria; n = 6 failed the GAPDH criteria; n = 3 failed the scaling factor criteria; and n = 1 failed both the GAPDH and scaling factor criteria.). The data from these 11 samples was compared to microarray data from n = 11 samples (matched for ancestry, age, gender, pack-years and pulmonary function test results) from the small airway epithelium of individuals with COPD that passed all QC criteria (see Additional file 1 for demographics of the 2 groups). The PCA revealed broad, global differences in genome-wide expression levels in the small airway epithelium of individuals with COPD in samples that pass QC vs those that fail (Figure 6). Using the criteria of P call of "Present" in 20% of samples, magnitude of fold-change in passed *vs *failed samples >1.5, and p < 0.01 using a t test with a Benjamini-Hochberg correction to limit the false positive rate, a total of 888 probe sets are differentially expressed between the 2 groups (Additional file 2), indicating that data from microarrays that fail QC criteria is not necessarily only more variable or "noisy," but in fact is significantly different biological data compared to data obtained from samples that pass QC criteria.

## Discussion

Epithelial samples (n = 223 total) of trachea, large airway and small airway were obtained from healthy subjects and from subjects with lung disease, including smokers and non-smokers, to assess quality control criteria for microarray analysis. Using Affymetrix Human Genome U133 Plus 2.0 arrays, a tripartite QC cutoff was established consisting of: (1) RNA quality, assessed by RNA Integrity Number (RIN) ≥ 7.0 using Agilent 2100 Bioanalyzer software; (2) cRNA transcript integrity, assessed by signal intensity ratio ≤ 3.0 of GAPDH 3' to 5' probe sets; and (3) the multi-chip normalization scaling factor ≤ 10.0. Of the 223 samples, 10 failed one or more of the QC criteria in a way that did not depend on phenotype of the subject or location of sampling. By using the QC cutoff criteria, the inter-array variability, as assessed by the coefficient of variation in the expression levels for 100 maintenance genes, decreased significantly. These QC criteria should be applicable to minimize experimental variation in gene expression microarray experiments.

### RNA Quality as Assessed by RIN

We have previously utilized the 28s/18s rRNA peak ratio, as calculated by electropherogram, to verify quality of RNA samples prior to microarray hybridization [38]. However, the 28s/18s ratio does not always provide a sufficient basis for distinguishing high quality from low quality RNA for microarray experiments [21,26,27,32,39-41]. For example, in an analysis of the effects of technical variability on gene expression in unfixed snap frozen *vs *formalin-fixed paraffin-embedded (FFPE) pelleted human bone marrow stromal cells, despite all RNA samples having equivalent and comparable 28s/18s ratios as visualized by computerized gel electrophoresis, more than twice as many genes were identified as expressed in snap frozen cells than in formalin-fixed paraffin-embedded cells, reflecting possible RNA quality effects in play that were not captured by quantitative assessment of the rRNA subunit peak heights [42].

Since the implementation of the Agilent Bioanalyzer RIN software, we have relied on the RIN as the primary indicator of RNA integrity, based on published data showing that the RIN accounts for numerous properties of the RNA degradation process to provide an unambiguous and comprehensive index of the overall quality of the starting material [21,41,43,44]. We found that the RNA quality in this study, as assessed by RIN, was generally good with a failure rate of 2.8% based on RIN ≥ 7.0. The low percentage of failures probably reflects rigorous training and standard operating procedures that ensure that epithelial cells are homogenized in Trizol in less than 60 minutes from the time of bronchial brushing. Using a single technician for this process with space, equipment and reagents that are not used for other purposes is also critical. The increased interest in using clinical specimens for research has led to widespread establishment of human tissue banks. In many cases, the RNA for microarray studies is extracted from tissues samples that may have been kept at room temperature and/or undergone repeated thawing and freezing, thereby affecting the quality of the RNA [24,32,45-47]. For example, microarray experiments involving pancreatic tumor tissue have had to discard the majority of the extracted RNA samples, due to the RNAse-rich content of the organ and the rapid degradation of the RNA material [48,49]. For those types of samples with possible RNA degradation, consistent application of the RIN ≥ 7.0 cutoff is useful for obtaining high quality gene expression microarray data.

Illustrating the predictive power of the RIN as a pre-chip criterion, linear regression modeling and ordinary least squares linear regression have shown that the scaling factor and GAPDH 3'/5' signal intensity ratio are negatively correlated with the RIN value [50]. Interestingly, the tandem failures by two samples in the present study by the RIN and scaling factor criteria, and by one sample by the RIN criterion and GAPDH 3'/5' signal intensity ratio criteria, are in concordance with the concept that poor RNA quality adversely affects synthesis of full-length cRNA as well as the hybridization efficiency of probe-target binding [19,33,34,39,50-52]. Since failure of the RIN test predicts failure at downstream steps, the application of this cutoff prior to *in vitro *transcription reactions and hybridization has the potential to save substantial costs in wasted reagents and technical time.

### Scaling

Published recommendations for an acceptable range of scaling factors computed at the same target intensity value vary in numerical fold cutoffs, or alternately, suggest all values within 2 standard deviations from the mean in either direction [16,34,35]. However, because the GeneChip Scanner 3000 7G used in this study, and generally employed by most institutional microarray core facilities, can resolve 65,535 levels of fluorescence in 16 bits of resolution (allowing for detection of very low levels of fluorescence), scaling factors for arrays can theoretically, and in practice, range well into the hundreds. Since a mutable scaling factor range can be continually subject to fluctuation as new samples are added to ongoing studies, and skewed by the presence of even one or two outlying chips with extremely high scaling factors, we chose a finite upper limit of 10.0 for the scaling factor criterion. As 97% of the scaling factor values for the samples examined in this study were ≤ 10.0, this is a practical and attainable cutoff that can accurately identify outlying poor quality samples.

In gene expression profiling studies of samples obtained from biopsies, cell sorting, or laser capture microdissection, yields of cellular RNA are often small quantities (e.g., ng) and require specialized amplification methods to generate sufficient biotinylated cRNA for array hybridization [53,54]. In these types of studies and others examining *in vivo *tissue from which sample RNA is limiting and alternate technical procedures are utilized, the scaling factor metric can be useful to assess the impact of technical artifact, and the quality of the expression data. For example, in an analysis of small sample RNAs from rat liver, significantly increased scaling factor values indicated that the amplification technique used contributed to technical variability in the form of a substantial decrease in the percent of transcripts detected on the array [55]. In contrast, in a study of small amounts of RNA derived from breast cancer tissue from mastectomy specimens, consistent scaling factor values across all amplified samples confirmed the validity and comparability of the expression data [56].

### Quality Control for Expression Microarray Analysis

Despite large amounts of published lung gene expression data, there is often little attention focused on microarray quality control, with the consequent risk of skewing the data by including poor quality arrays in the analysis [35,57-63]. Further, different effects of RNA quality on specific ontological categories can complicate the extraction of biological information from microarrays of varying quality. For example, in an analysis of the effects of RNA integrity on gene expression in breast cancer samples, it was found that specific categories of genes such as those related to deoxyribonuclease activity, regulation of cell adhesion, and NADH dehydrogenase activity, were most affected by RNA quality [26].

One methodology for testing data integrity is that of unsupervised hierarchical sample clustering based on Spearman correlations-based distance metric [64,65]. The resulting clusters are inspected manually for clustering of samples by non-biological parameters, such as the dates of sample collection and RNA extraction, the batch of *in vitro *transcription and amplification reagent used, and the date of array hybridization. These factors may contribute to batch effects, where the overall intensity of a batch of microarrays more closely resembles the batch than the rest of the group of arrays [60,66]. While these clustering methods provide insight into experimental variability, they provide no quantitative guidelines for eliminating microarrays from analysis and it is sometimes difficult to determine if the clusters have any relationship to biological variability.

Another strategy often used for differentiating high quality from low quality microarray data is based on outlier status of any given sample in an experiment. Software packages such as dChip (DNA-chip analyzer) and Probe Profiler can identify intensity outliers of a sample in a group of microarrays, and take into account such features of the array hybridization such as brightness, saturation, dynamic range, and background [65-67]. The caveat is that all chips must be from biologically comparable origins and that only a small number of experimental outliers must exist.

### QC Criteria Presented in this Study

The current study provides an efficient and simple approach for quality assessment of gene expression microarray data. It emphasizes good experimental execution and discarding unsatisfactory microarrays rather than salvaging data through complex statistical analyses of array data of variable quality. We provide a standardized tripartite criteria specifically addressing starting RNA quality, integrity of the cRNA transcript, and hybridization efficiency. Each parameter has been assigned a threshold value, outside of which samples are readily identifiable as being low quality and can be eliminated or re-hybridized before proceeding to analysis. All measures are available through Agilent Bioanalyzer software and the Affymetrix GCOS report automatically generated after array washing and scanning. Although the Agilent Bioanalyzer and Affymetrix platforms are widely used, analogous criteria may be applied for alternate methodologies. For example, assessment of the relative signal for probes representing the 3' and 5' ends of any mRNA could be included as QC for any microarray platform.

In the context of data sharing via public repositories, the criteria presented in this study has the benefit of including two parameters that are guaranteed to be available for any Affymetrix data deposited in GEO. The initial processing by GCOS of CEL files produces a Quality Report containing the 3'/5' GAPDH signal intensity ratio and a multi-chip normalization scaling factor for the array. The GCOS software is available for free download from Affymetrix and can be applied by all investigators. In this way, two of the three QC criteria discussed in this paper provide a consistent quality control approach to not only current data, but also to previously published, archived data. Even though the RIN criterion as applied here requires specialized equipment and software, the RIN can be indirectly predicted from the 3'/5' ratio which is extracted from the CEL files deposited in GEO [50].

## Conclusion

In the context that minimizing undesirable technical variation allows for more accurate analysis of gene expression and increased power for significance testing, we propose that the simple method described here, consisting of a universally available set of three criteria, can ensure that microarray data reflects biological differences as opposed to experimental variability.

## Methods

### Study Population

After signing informed consent, subjects were evaluated in the Weill Cornell NIH Clinical and Translational Science Center and Department of Genetic Medicine Clinical Research Facility under protocols approved by the Weill Cornell Medical College Institutional Review Board. All individuals were assessed by standard history, physical exam, complete blood count, coagulation studies, liver function tests, HIV-1 test, urine studies, chest X-ray, EKG, and pulmonary function tests. All individuals were assessed for smoking status with urine nicotine and cotinine levels, and blood carboxyhemoglobin levels. A total of 223 airway epithelial samples in this study were derived from three sites: trachea, large airway (2^nd^-3^rd ^order bronchi) and small airway (10^th^-12^th ^order bronchi) in five phenotypic groups (Table 1): healthy non-smokers (trachea chea n = 17, large airway n = 21, small airway n = 35), healthy smokers (trachea n = 15, large airway n = 32, small airway n = 44), symptomatic smokers (trachea n = 3, large airway n = 4, small airway n = 10), smokers with lone emphysema with normal spirometry (small airway n = 22) and smokers with COPD GOLD stages I-III (small airway n = 20). Healthy non-smokers had no symptoms referable to the lungs, normal lung function and normal chest X-ray, and all laboratory tests within normal limits. The criteria for healthy smokers were identical to that of healthy non-smokers except urine nicotine and cotinine and blood carboxyhemoglobin levels confirmed current smoking status. Symptomatic smokers were similar to healthy smokers except they had cough or sputum score of 3 or greater, or dyspnea score on the Modified Medical Research Council (MMRC) dyspnea scale of 2 or greater [68-71]. The lone emphysema with normal spirometry phenotype was defined by normal FEV1/FVC, reduced DLCO, and evidence of emphysema on quantitative CT scan (>1% of lung with <-950 Hounsfield units [30]). Smokers with established COPD included current smokers who met the Global Initiative for Chronic Obstructive Lung Disease (GOLD) criteria for GOLD I, II, and III [72]. An independent data set from n = 11 individuals with COPD was available from a technician training program in the Weill Cor-nell Medical College Department of Genetic Medicine. The small airway epithelium samples from these subjects, which failed QC criteria, were compared to 11 matched small airway epithelium samples from individuals with COPD that passed QC criteria (see Additional file 1).

### Sampling of Airway Epithelium and RNA extraction

Fiberoptic bronchoscopy was performed to obtain pure populations of tracheal, large and small airway epithelium by using methods previously described [4,29,73]. Briefly, after mild sedation with meperidine and midazolam and routine anesthesia of the vocal cords and bronchial airways with topical lidocaine, a fiberoptic bronchoscope (Pentax, EB-1530T3) was taken proximal to desired collection location. A 2.0 mm disposable brush is used for brushing immediately distal to the location of the bronchscope (for trachea or large airway) or by advancing 7 to 10 cm further into the 10^th ^to 12^th ^generation branching for small airway. Epithelium was collected by gently gliding the brush back and forth 5 to 10 times in 8 to 10 different locations in the same general area. Cells were detached by immersing the brush into 5 ml of ice-cold bronchial epithelial basal medium (BEBM, Clonetics, Walkersville, MD) and flicking five to ten times. An aliquot of 0.5 ml was used for differential cell count and the remainder (4.5 ml) was centrifuged at 6,000 rpm for 10 minutes within less than 60 minutes from the time of bronchial brushing. Pelleted airway epithelial cells were lysed with the TRIzol reagent (InVitrogen, Carlsbad, CA), and after chloroform extraction the RNA was purified directly from the aqueous phase using the RNeasy MinElute RNA isolation kit (Qiagen, Valencia, CA). For each sample, 1 μl of RNA was used for quantification of yield by NanoDrop ND-1000 spectrophotometer (NanoDrop Technologies, Wilmington, DE) and quality assessment by Agilent 2100 Bioanalyzer software. The samples were stored in RNA Secure (Ambion, Austin, TX) at -80°C until time of biotin-labeled cRNA preparation.

### Microarray Processing

Double stranded cDNA was synthesized from 1.0 to 2.0 μg of total RNA using the GeneChip One-Cycle cDNA Synthesis Kit, followed by cleanup of the double stranded product with the GeneChip Sample Cleanup Module. The GeneChip IVT Labeling Kit was used for the 16 hr *in vitro *transcription reaction and the Genechip Sample Cleanup Module was used for cleanup of the biotin-labeled cRNA (all kits from Affymetrix, Santa Clara, CA). Final yield of biotin-labeled cRNA was confirmed by NanoDrop spectrophotometric analysis. For each sample, 10 μg of biotin-labeled cRNA was fragmented, and hybridized to the Human Genome U133 Plus 2.0 array (54,675 probe sets) according to Affymetrix protocols, processed by the Affymetrix GeneChip Fluidics Station 450 and scanned with an Affymetrix GeneChip Scanner 3000 7G , as previously described [4]. Captured images were analyzed using Microarray Suite version 5.0 (MAS 5.0) algorithm (Affymetrix). The data was normalized per array using GeneSpring version 7.3 software (Agilent Technologies, Palo Alto, CA), by dividing the raw data by the 50^th ^percentile of all measurements on that array. All microarray data has been deposited at the Gene Expression Omnibus (GEO) site (; accession number GSE11906).

### Quality Control Parameters

The selection of the three QC criteria was targeted towards addressing quality control in the three integral stages of the microarray process: (1) extraction of the starting RNA material; (2) synthesis of cDNA and antisense biotin-labeled cRNA target; and (3) the array hybridization efficiency.

#### RIN

An RNA Integrity Number (RIN) for each RNA sample in this study was generated by an Agilent Bioanalyzer algorithm that uses a Bayesian approach to train and select a prediction model incorporating features extracted from an electropherogram including pre-region, 5S-region, fast-region, 18S-fragment, inter-region, 28S-fraction, precursor-region, and post-region [41,44]. RIN values range from 1 to 10, with 1 indicating a high level of degradation and 10 indicating fully intact RNA. RIN was assessed on 180 of 223 epithelial RNA samples. The 43 RNA samples not assessed by RIN had been processed and hybridized to microarray before the development of the RIN software and residual RNA was unavailable for testing. Published suggestions of a RIN cutoff value to distinguish poor quality from good quality RNA samples vary from 3.9 to 7.8 [19,26,32,40,50,74]. Based on literature indicating a substantial increase in the rate of false positives on the array when the starting RNA had a RIN value of <7.0, an acceptance criterion of RIN ≥ 7.0 was established [33]. Available RIN values for the 180 RNA preparations were assessed by this criteria and passing or failing samples were grouped by phenotype, and each phenotype was separated by biologic origin.

#### GAPDH 3'/5' signal intensity ratio

Per Affymetrix guidelines, the ratio of the 3' to 5' signal intensity values can be used as a method of quality control for the array data [23,35,36,75]. As the GeneChip system utilizes polyadenylation complementary oligonucleotides as a primer for reverse transcription of the starting RNA template, inefficiency of first strand cDNA synthesis and/or *in vitro *transcription of cRNA can result in under representation of the 5' moiety of the transcript [34,52]. In accordance with recommendations by Affymetrix and others, an acceptance criterion of GAPDH 3'/5' ratio ≤ 3.0 was established [16,34,35]. To accomplish this, for each sample hybridized to microarray, a GeneChip Operating Software report file was generated using the Affymetrix GeneChip Operating Software (GCOS), a software system that automates the acquisition of data by GeneChip fluidics stations and scanners, and provides workflow tracking of experiment, image and analysis data. Among the QC metrics summarized in the report file are the signal intensity values for the 3' and 5' probe sets for the GAPDH gene. The ratio of 3' to 5' signal intensities for the GAPDH probe set was extracted from the GCOS report file for each of 223 samples and those with GAPDH 3'/5' ratio > 3.0 were scored as failures.

#### Multi-chip normalization scaling factor

According to Affymetrix microarray guidelines, comparable scaling factors between arrays in a given experiment are critical to minimizing differences in overall signal intensities, thereby allowing for more reliable detection of biologically relevant changes [35,52]. Based on the distribution of data for 223 samples, a criterion of scaling factor ≤ 10.0 was established, above which samples were considered to demonstrate poor hybridization and labeling efficiency. Scaling factor values for all 223 samples were assessed against this acceptable level. To accomplish this, for each sample hybridized to a microarray, the Affymetrix GeneChip Scanner 3000 7G was set to a target intensity value of 500 and the GCOS image analysis software extracted pixel values from the raw image file, producing a CEL file containing fluorescence intensities for each probe. A CHP file was then generated from each CEL file through GCOS consolidation of all probe pairs interrogating a gene into a single signal value and an Absent/Marginal/Present call for the probe set. The creation of CHP files from CEL files generated a scaling factor for each array which was applied to normalize signal intensity thereby permitting comparisons among arrays. The scaling factor is the multiplication factor applied to the trimmed mean of probe set intensities to equalize this value to the target intensity value. Scaling factor values were extracted from the GCOS report file for all 223 samples.

### Analysis of Maintenance Gene Expression Levels

Samples that failed any one of the three criteria described were considered to have failed the QC criteria, while those samples that passed all three criteria were considered to have passed the QC criteria. To confirm the validity of this quality assessment strategy, expression levels were determined for a set of 100 constitutively expressed maintenance genes and differences in gene expression profile for these genes were compared between the samples failing the quality control criteria and the samples passing the QC criteria. The set of control genes was selected by Affymetrix using the *a priori *knowledge that they exhibit relatively low signal variation over different sample types and are consistently called Present in a large number of different tissues and cell lines (list available at the NetAffx Analysis Center, ). In the present study, for notational convenience, we use the term "gene" in place of "probe set", as each one of the 100 probe sets represents a different gene.

### Statistical Analysis

To examine potential causes for variation in QC criterion values between samples, the effects of differences in phenotype or biologic origin of the sample were assessed by ANOVA.

In regard to the maintenance genes we used Pearson's correlation to assess correlation in expression levels for the 100 maintenance genes among the 10 samples (2 large airway epithelium, 8 small airway epithelium) that failed the QC criteria and 24 randomly selected samples that passed QC criteria (8 tracheal epithelium, 8 large airway epithelium, and 8 small airway epithelium; see Results) [76]. The identities of the 24 samples passing QC criteria were randomly generated using a random number generator and sample origin was evenly distributed among trachea (n = 8), large airway (n = 8), and small airway (n = 8). Significance of the difference in correlation coefficients for pairwise correlations where at least one sample failed QC criteria and for pairwise correlations where both samples passed QC criteria was assessed by nonparametric analysis. Coefficient of variation analysis was used to determine variability in expression levels for each of the 100 maintenance genes across the 10 samples failing QC criteria and across 10 samples passing QC criteria. For this analysis, the 10 samples passing QC criteria were randomly selected using a random number generator, with the stipulation that these samples were matched in origin with the 10 samples failing QC criteria (i.e., 2 large airway epithelium, 8 small airway epithelium). For the purposes of the coefficient of variation analysis, the data set of 10 samples passing QC criteria was termed "pass", and the data set of 10 samples failing QC criteria was termed "fail". Significance of the difference in coefficients of variation of gene expression levels across the "pass" and "fail" data sets was determined by Mann-Whitney *U *test.

To compare gene expression profiles in samples that passed QC to those that failed QC, principal components analysis was carried out using Partek^® ^Genomics Suite software (version 6.8 Copyright^© ^2008) for 11 COPD subjects who failed chip quality control and 11 COPD subjects who passed (matched for gender, age, ethnicity, and smoking history). Affymetrix HG-U133 Plus 2.0 CEL files were imported into Partek using the Robust Multi-chip Average (RMA) method. All 54,675 log2-transformed small airway gene expression data were mapped to principal components to preserve the variation of this data, projected in 3 dimensions, and plotted. In order to identify the specific probe sets that were differentially expressed between the two groups, microarray data were processed using the MAS5 algorithm (Affymetrix Microarray Suite Version 5 software), which takes into account the perfect match and mismatch probes. MAS5-processed data were normalized using GeneSpring by setting measurements <0.01 to 0.01 and by normalizing per chip to the median expression value on that array and, per gene to the median expression value for each gene across all arrays. Genes that were significantly differentially expressed between the two groups were selected according to the following criteria: (1) P call of "Present" in 20% of samples; (2) magnitude of fold change in average expression value for pass QC vs fail QC of >1.5; and (3) p < 0.01 using a t test with a Benjamini-Hochberg correction to limit the false positive rate [77].

## Authors' contributions

All authors have read and approved the final version of the manuscript. **TR **conducted the microarray experiments, performed the analysis and helped draft the manuscript. **TO **and **NH **participated in study design and statistical analysis, supervised the data collection and helped draft the manuscript. **WW **assisted with data analysis and study design **BGH **coordinated the collection of all of the clinical samples used in the microarray analysis **MA, DD, **and **MT **processed samples and conducted microarray experiments **RGC **conceived of the study, and assisted in study design and helped to draft the manuscript.

## Supplementary Material

Additional file 1 **Demographics of the Study Population and Biologic Samples for the Comparison of Fail QC or Pass QC Data**. All subjects from both groups were smokers with COPD. The "passed" and "failed" groups are comprised of samples that passed or failed, respectively, the QC criteria. Data is presented as mean ± standard deviation. Click here for file

Additional file 2 **Significant Genes in the Small Airways Epithelium of Smokers with COPD Between Chips that Failed QC and Chips that Passed QC**. Shown are the 888 probe sets that are differentially expressed (using criteria of a fold change greater than 1.5 and a p value, with Benjamini-Hochberg correction, less than 0.01, in n = 11 pass QC samples and n = 11 fail QC samples, all from the small airway epithelium of individuals with COPD. Click here for file
