# Supplementary material for: Quality control in microarray assessment of gene expression in human airway epithelium
Source: BMC Genomics. 2009 Oct 24;10:493. doi: 10.1186/1471-2164-10-493 (PMC2774870; doi:10.1186/1471-2164-10-493)
Supplement: Additional file 1 — Demographics of the Study Population and Biologic Samples for the Comparison of Fail QC or Pass QC Data. All subjects from both groups were smokers with COPD. The "passed" and "failed" groups are comprised of samples that passed or failed, respectively, the QC criteria. Data is presented as mean ± standard deviation. [file 1471-2164-10-493-S1.PDF]

Additional File 1. Demographics of the Study Population and Biologic Samples for the Comparison of Fail QC or Pass QC Data[1]

Parameter	Small airway	
	COPD (Passed)	COPD (Failed)
n	11	11
Age	51 ± 6	54 ± 5
Gender[2]	9/2	7/4
Ancestry[3]	6/2/0/3	7/2/0/2
Smoking history	29 ± 14	31 ± 14
Pulmonary function parameters[4]		
FVC	101 ± 13	114 ± 4
FEV1	76 ± 13	81 ± 12
FEV1/FVC	61 ± 7	58 ± 9
TLC	103 ± 22	117 ± 8
DLCO	78 ± 13	77 ± 16
Total # of cells recovered ($x10^6$)	6.9	5.2
Cell differential[5]		
% epithelial	99 ± 1	98 ± 2
% inflammatory	1 ± 1	2 ± 2
% ciliated	61 ± 13	56 ± 16
% secretory	12 ± 6	15 ± 5
% basal	14 ± 7	15 ± 9
% undifferentiated	13 ± 4	13 ± 4

1 All subjects from both groups were smokers with COPD. The "passed" and "failed" groups are comprised of samples that passed or failed, respectively, the QC criteria. Data is presented as mean ± standard deviation.

2 Male/Female.

3 European/Hispanic/Asian/African.

4 Pulmonary function testing parameters are given as percent of predicted value with the exception of FEV1/FVC, which is reported as % observed; FVC - forced vital capacity, FEV1 - forced expiratory volume in 1 sec, TLC - total lung capacity, DLCO - diffusing capacity. For individuals with COPD, FVC, FEV1, and FEV1/FVC are post-bronchodilator values.

5 % epithelial and inflammatory are based on the total number of cells recovered; % ciliated, secretory, basal and undifferentiated cells are based on the total number of epithelial cells recovered.
