# Supplementary material for: Quality control in microarray assessment of gene expression in human airway epithelium
Source: BMC Genomics. 2009 Oct 24;10:493. doi: 10.1186/1471-2164-10-493 (PMC2774870; doi:10.1186/1471-2164-10-493)
Supplement: Additional file 2 — Significant Genes in the Small Airways Epithelium of Smokers with COPD Between Chips that Failed QC and Chips that Passed QC. Shown are the 888 probe sets that are differentially expressed (using criteria of a fold change greater than 1.5 and a p value, with Benjamini-Hochberg correction, less than 0.01, in n = 11 pass QC samples and n = 11 fail QC samples, all from the small airway epithelium of individuals with COPD. [file 1471-2164-10-493-S2.PDF]

Additional File 2. Significant Genes in the Small Airways Epithelium of Smokers with COPD Between Chips that Failed QC and Chips that Passed QC[1]

Probe set ID	Gene symbol	Gene title	Fold-change (Fail QC/Pass QC)	p value
1559361_at	7A5	putative binding protein 7a5	-4.345	$2.89x10^{-03}$
1554878_a_at	ABCD3	ATP-binding cassette, sub-family D (ALD), member 3	-3.012	$1.25x10^{-03}$
222697_s_at	ABHD10	abhydrolase domain containing 10	-2.203	$3.86x10^{-03}$
205566_at	ABHD2	abhydrolase domain containing 2	-2.162	$9.31x10^{-03}$
221815_at	ABHD2	abhydrolase domain containing 2	-2.834	$5.51x10^{-03}$
63825_at	ABHD2	abhydrolase domain containing 2	-2.350	$1.76x10^{-03}$
87100_at	ABHD2	abhydrolase domain containing 2	-5.957	$1.24x10^{-04}$
209856_x_at	ABI2	abl interactor 2	-1.680	$5.17x10^{-03}$
210461_s_at	ABLIM1	actin binding LIM protein 1	-3.139	$3.86x10^{-03}$
201629_s_at	ACP1	acid phosphatase 1, soluble	-3.733	$2.21x10^{-04}$
215227_x_at	ACP1	acid phosphatase 1, soluble	-2.293	$6.36x10^{-03}$
1554390_s_at	ACTR2	ARP2 actin-related protein 2 homolog (yeast)	-3.420	$1.00x10^{-03}$
1558015_s_at	ACTR2	ARP2 actin-related protein 2 homolog (yeast)	-3.155	$3.93x10^{-03}$
202604_x_at	ADAM10	ADAM metallopeptidase domain 10	-2.283	$1.64x10^{-04}$
1555326_a_at	ADAM9	ADAM metallopeptidase domain 9 (meltrin gamma)	-3.569	$5.35x10^{-03}$
239439_at	AFF4	AF4/FMR2 family, member 4	-2.433	$5.90x10^{-03}$
208042_at	AGGF1	angiogenic factor with G patch and FHA domains 1	-2.038	$6.99x10^{-03}$
200848_at	AHCYL1	S-adenosylhomocysteine hydrolase-like 1	-4.180	$2.60x10^{-04}$
220290_at	AIM1L	absent in melanoma 1-like	1.989	$8.80x10^{-04}$
241326_at	AK7	adenylate kinase 7	-2.560	$1.92x10^{-04}$
208325_s_at	AKAP13	A kinase (PRKA) anchor protein 13	-1.814	$7.76x10^{-03}$
202053_s_at	ALDH3A2	aldehyde dehydrogenase 3 family, member A2	-2.195	$3.93x10^{-03}$
210544_s_at	ALDH3A2	aldehyde dehydrogenase 3 family, member A2	-3.285	$1.54x10^{-03}$
231973_s_at	ANAPC1	anaphase promoting complex subunit 1	-2.126	$3.20x10^{-04}$
208721_s_at	ANAPC5	anaphase promoting complex subunit 5	-3.408	$1.56x10^{-04}$
233292_s_at	ANKHD1 /// ANKHD1-EIF4EBP3 /// EIF4EBP3	ankyrin repeat and KH domain containing 1 /// ANKHD1-EIF4EBP3 /// eukaryotic translation initiation factor 4E binding protein 3	-2.969	$2.54x10^{-04}$
223532_at	ANKRD39	ankyrin repeat domain 39	-1.825	$2.60x10^{-03}$
200612_s_at	AP2B1	adaptor-related protein complex 2, beta 1 subunit	-1.619	$9.79x10^{-03}$
203141_s_at	AP3B1	adaptor-related protein complex 3, beta 1 subunit	-2.164	$9.14x10^{-04}$
203526_s_at	APC	adenomatous polyposis coli	-2.176	$6.16x10^{-04}$

Additional File 2. Significant Genes in the Small Airways Epithelium of Smokers with COPD Between Chips that Failed QC and Chips that Passed QC[1] (cont., page 2)

Probe set ID	Gene symbol	Gene title	Fold-change (Fail QC/Pass QC)	p value
214995_s_at	APOBEC3F /// APOBEC3G	apolipoprotein B mRNA editing enzyme, catalytic polypeptide-like 3F /// apolipoprotein B mRNA editing enzyme, catalytic polypeptide-like 3G	-2.484	9.65×10^{-03}
202631_s_at	APPBP2	amyloid beta precursor protein (cytoplasmic tail) binding protein 2	-1.606	9.14×10^{-04}
39249_at	AQP3	aquaporin 3 (Gill blood group)	-3.185	5.34×10^{-03}
208750_s_at	ARF1	ADP-ribosylation factor 1	-3.182	3.78×10^{-03}
202955_s_at	ARFGEF1	ADP-ribosylation factor guanine nucleotide-exchange factor 1(brefeldin A-inhibited)	-2.095	8.08×10^{-03}
222508_s_at	ARGLU1	arginine and glutamate rich 1	-6.977	3.26×10^{-03}
230803_s_at	ARHGAP24	Rho GTPase activating protein 24	-1.838	7.46×10^{-03}
1552627_a_at	ARHGAP5	Rho GTPase activating protein 5	-2.832	9.50×10^{-04}
201878_at	ARIH1	ariadne homolog, ubiquitin-conjugating enzyme E2 binding protein, 1 (Drosophila)	-1.943	2.60×10^{-04}
201879_at	ARIH1	ariadne homolog, ubiquitin-conjugating enzyme E2 binding protein, 1 (Drosophila)	-2.316	8.23×10^{-03}
201229_s_at	ARIH2	ariadne homolog 2 (Drosophila)	-2.070	1.25×10^{-03}
201657_at	ARL1	ADP-ribosylation factor-like 1	-1.918	4.46×10^{-03}
202092_s_at	ARL2BP	ADP-ribosylation factor-like 2 binding protein	-1.990	5.51×10^{-03}
200760_s_at	ARL6IP5	ADP-ribosylation-like factor 6 interacting protein 5	-2.241	1.27×10^{-03}
210971_s_at	ARNTL	aryl hydrocarbon receptor nuclear translocator-like	-2.835	4.23×10^{-05}
214553_s_at	ARPP-19	cyclic AMP phosphoprotein, 19 kD	-1.938	3.93×10^{-03}
203428_s_at	ASF1A	ASF1 anti-silencing function 1 homolog A (S. cerevisiae)	-2.219	3.96×10^{-03}
1554980_a_at	ATF3	activating transcription factor 3	-2.630	5.80×10^{-04}
218987_at	ATF7IP	activating transcription factor 7 interacting protein	-1.810	7.22×10^{-04}
231825_x_at	ATF7IP	activating transcription factor 7 interacting protein	1.776	7.43×10^{-03}
213026_at	ATG12	ATG12 autophagy related 12 homolog (S. cerevisiae)	-1.598	9.45×10^{-03}
210639_s_at	ATG5	ATG5 autophagy related 5 homolog (S. cerevisiae)	-2.188	6.96×10^{-03}
212362_at	ATP2A2	ATPase, Ca++ transporting, cardiac muscle, slow twitch 2	-1.757	6.03×10^{-04}
1554177_a_at	ATP5S	ATP synthase, H+ transporting, mitochondrial F0 complex, subunit s (factor B)	1.759	7.20×10^{-03}
201971_s_at	ATP6V1A	ATPase, H+ transporting, lysosomal 70kDa, V1 subunit A	-3.537	3.13×10^{-03}
202873_at	ATP6V1C1	ATPase, H+ transporting, lysosomal 42kDa, V1 subunit C1	-2.227	9.20×10^{-03}

Additional File 2. Significant Genes in the Small Airways Epithelium of Smokers with COPD Between Chips that Failed QC and Chips that Passed QC[1] (cont., page 3)

Probe set ID	Gene symbol	Gene title	Fold-change (Fail QC/Pass QC)	p value
211852_s_at	ATRN	attractin	-1.889	$8.02x10^{-03}$
1562391_at	B3GALNT2	beta-1,3-N-acetylgalactosaminyltransferase 2	2.027	$5.59x10^{-03}$
1554835_a_at	B3GNT5	UDP-GlcNAc:betaGal beta-1,3-N-acetylglucosaminyltransferase 5	-4.782	$6.73x10^{-05}$
210818_s_at	BACH1	BTB and CNC homology 1, basic leucine zipper transcription factor 1	-2.749	$8.37x10^{-03}$
212744_at	BBS4	Bardet-Biedl syndrome 4	-1.682	$3.23x10^{-03}$
212745_s_at	BBS4	Bardet-Biedl syndrome 4	-2.164	$2.03x10^{-03}$
206665_s_at	BCL2L1	BCL2-like 1	-3.419	$1.11x10^{-03}$
224035_s_at	BCL2L13	BCL2-like 13 (apoptosis facilitator)	-3.081	$6.93x10^{-03}$
215990_s_at	BCL6	B-cell CLL/lymphoma 6	-3.073	$3.01x10^{-03}$
201101_s_at	BCLAF1	BCL2-associated transcription factor 1	-4.428	$6.16x10^{-04}$
214499_s_at	BCLAF1	BCL2-associated transcription factor 1	-2.411	$9.90x10^{-03}$
210214_s_at	BMPR2	bone morphogenetic protein receptor, type II (serine/threonine kinase)	-2.783	$2.88x10^{-03}$
216521_s_at	BRCC3	BRCA1/BRCA2-containing complex, subunit 3	-3.131	$5.73x10^{-04}$
208685_x_at	BRD2	bromodomain containing 2	-1.910	$6.94x10^{-03}$
222737_s_at	BRD7	bromodomain containing 7	-2.578	$4.97x10^{-04}$
201235_s_at	BTG2	BTG family, member 2	-2.309	$2.74x10^{-03}$
224667_x_at	C10orf104	chromosome 10 open reading frame 104	1.589	$7.40x10^{-03}$
210455_at	C10orf28	chromosome 10 open reading frame 28	-7.854	$2.71x10^{-03}$
220919_s_at	C10orf79	chromosome 10 open reading frame 79	-5.174	$1.80x10^{-04}$
243896_at	C10orf79	chromosome 10 open reading frame 79	-3.180	$1.64x10^{-03}$
222767_s_at	C12orf49	chromosome 12 open reading frame 49	-1.819	$5.97x10^{-03}$
1560426_at	C12orf55	chromosome 12 open reading frame 55	-2.993	$6.99x10^{-03}$
214264_s_at	C14orf143	chromosome 14 open reading frame 143	-1.757	$9.23x10^{-03}$
232814_x_at	C14orf153	Chromosome 14 open reading frame 153	1.774	$9.99x10^{-03}$
225088_at	C16orf63	chromosome 16 open reading frame 63	-2.090	$1.88x10^{-03}$
232972_at	C17orf72	chromosome 17 open reading frame 72	-2.299	$2.26x10^{-03}$
223352_s_at	C17orf80	chromosome 17 open reading frame 80	-2.719	$2.36x10^{-03}$
225863_s_at	C19orf12	chromosome 19 open reading frame 12	-2.015	$8.91x10^{-03}$
212574_x_at	C19orf6	chromosome 19 open reading frame 6	-5.990	$5.84x10^{-04}$
213986_s_at	C19orf6	chromosome 19 open reading frame 6	-5.074	$7.46x10^{-03}$
243753_at	C1orf173	chromosome 1 open reading frame 173	-2.967	$4.48x10^{-03}$
233750_s_at	C1orf25	chromosome 1 open reading frame 25	-2.543	$7.22x10^{-04}$
224693_at	C20orf108	chromosome 20 open reading frame 108	-1.598	$1.69x10^{-03}$
1554818_s_at	C20orf12	chromosome 20 open reading frame 12	-4.259	$7.31x10^{-05}$
1554657_a_at	C20orf26	chromosome 20 open reading frame 26	-3.116	$2.76x10^{-03}$
240453_at	C20orf26	chromosome 20 open reading frame 26	-3.156	$1.33x10^{-03}$
236390_at	C20orf94	chromosome 20 open reading frame 94	-3.711	$9.88x10^{-03}$
220941_s_at	C21orf91	chromosome 21 open reading frame 91	-1.714	$7.58x10^{-03}$
219662_at	C2orf49	chromosome 2 open reading frame 49	-1.723	$6.49x10^{-03}$
1563814_at	C2orf50	chromosome 2 open reading frame 50	-6.266	$2.82x10^{-03}$

Additional File 2. Significant Genes in the Small Airways Epithelium of Smokers with COPD Between Chips that Failed QC and Chips that Passed QC[1] (cont., page 4)

Probe set ID	Gene symbol	Gene title	Fold-change (Fail QC/Pass QC)	p value
1554147_s_at	C3orf15	chromosome 3 open reading frame 15	-2.276	$6.59x10^{-03}$
1556487_a_at	C3orf15	chromosome 3 open reading frame 15	-3.195	$2.33x10^{-04}$
1561429_a_at	C3orf15	chromosome 3 open reading frame 15	-3.945	$3.72x10^{-03}$
1561430_s_at	C3orf15	chromosome 3 open reading frame 15	-3.316	$1.64x10^{-04}$
1561928_s_at	C3orf16	chromosome 3 open reading frame 16	-2.203	$5.73x10^{-04}$
226524_at	C3orf38	chromosome 3 open reading frame 38	-2.111	$5.68x10^{-03}$
1554229_at	C5orf41	chromosome 5 open reading frame 41	-2.272	$2.03x10^{-03}$
232777_s_at	C6orf118	chromosome 6 open reading frame 118	-2.520	$5.84x10^{-04}$
227656_at	C6orf70	chromosome 6 open reading frame 70	-1.732	$5.33x10^{-03}$
225576_at	C6orf72	chromosome 6 open reading frame 72	-2.264	$1.77x10^{-03}$
225600_at	C8orf83	chromosome 8 open reading frame 83	-1.975	$7.15x10^{-03}$
225914_s_at	CAB39L	calcium binding protein 39-like	-1.660	$6.47x10^{-04}$
200756_x_at	CALU	calumenin	-2.458	$9.39x10^{-04}$
203357_s_at	CAPN7	calpain 7	-2.501	$4.17x10^{-03}$
200722_s_at	CAPRIN1	cell cycle associated protein 1	-2.580	$1.48x10^{-03}$
202402_s_at	CARS	cysteinyl-tRNA synthetase	-2.128	$3.86x10^{-03}$
240983_s_at	CARS	cysteinyl-tRNA synthetase	-7.196	$2.95x10^{-03}$
207686_s_at	CASP8	caspase 8, apoptosis-related cysteine peptidase	-3.344	$2.59x10^{-03}$
210775_x_at	CASP9	caspase 9, apoptosis-related cysteine peptidase	-1.716	$1.56x10^{-03}$
1559409_a_at	CC2D2A	coiled-coil and C2 domain containing 2A	-6.198	$5.84x10^{-04}$
1554513_s_at	CCDC123	coiled-coil domain containing 123	-3.925	$6.93x10^{-04}$
229082_at	CCDC125	coiled-coil domain containing 125	-3.350	$2.34x10^{-05}$
228061_at	CCDC126	coiled-coil domain containing 126	-2.077	$2.93x10^{-03}$
1554145_a_at	CCDC128	coiled-coil domain containing 128	-1.925	$7.08x10^{-03}$
211559_s_at	CCNG2	cyclin G2	-3.641	$7.15x10^{-03}$
221427_s_at	CCNL2	cyclin L2	-6.608	$5.91x10^{-03}$
201946_s_at	CCT2	chaperonin containing TCP1, subunit 2 (beta)	-3.322	$3.75x10^{-06}$
209772_s_at	CD24	CD24 molecule	-3.343	$8.50x10^{-03}$
207549_x_at	CD46	CD46 molecule, complement regulatory protein	-2.600	$2.06x10^{-03}$
211574_s_at	CD46	CD46 molecule, complement regulatory protein	-2.527	$2.44x10^{-03}$
217881_s_at	CDC27	cell division cycle 27 homolog (S. cerevisiae)	-1.942	$3.78x10^{-03}$
211289_x_at	CDC2L1 /// CDC2L2	cell division cycle 2-like 1 (PITSLRE proteins) /// cell division cycle 2-like 2 (PITSLRE proteins)	-2.800	$5.96x10^{-04}$
207319_s_at	CDC2L5	cell division cycle 2-like 5 (cholinesterase-related cell division controller)	-2.261	$3.91x10^{-04}$
209055_s_at	CDC5L	CDC5 cell division cycle 5-like (S. pombe)	-3.984	$5.40x10^{-04}$
201130_s_at	CDH1	cadherin 1, type 1, E-cadherin (epithelial)	-5.644	$6.77x10^{-03}$
233662_at	CDH26	cadherin-like 26	-2.386	$7.46x10^{-03}$
207766_at	CDKL1	cyclin-dependent kinase-like 1 (CDC2-related	-5.111	$3.23x10^{-03}$

Additional File 2. Significant Genes in the Small Airways Epithelium of Smokers with COPD Between Chips that Failed QC and Chips that Passed QC[1] (cont., page 5)

Probe set ID	Gene symbol	Gene title	Fold-change (Fail QC/Pass QC)	p value
		kinase)		
244698_at	CDRT4	CMT1A duplicated region transcript 4	-2.416	$5.33x10^{-03}$
212746_s_at	CEP170	centrosomal protein 170kDa	-2.239	$4.70x10^{-03}$
1554489_a_at	CEP70	centrosomal protein 70kDa	-2.003	$9.23x10^{-03}$
224600_at	CGGBP1	CGG triplet repeat binding protein 1	-1.882	$6.02x10^{-03}$
223232_s_at	CGN	cingulin	-2.581	$3.71x10^{-03}$
207645_s_at	CHD1L	chromodomain helicase DNA binding protein 1-like	-2.463	$1.34x10^{-03}$
201183_s_at	CHD4	chromodomain helicase DNA binding protein 4	-2.634	$2.60x10^{-04}$
235388_at	CHD9	chromodomain helicase DNA binding protein 9	-10.190	$2.76x10^{-03}$
204233_s_at	CHKA	choline kinase alpha	-2.975	$8.06x10^{-03}$
1555278_a_at	CKAP5	cytoskeleton associated protein 5	-2.415	$5.64x10^{-03}$
1555543_a_at	CLCC1	chloride channel CLIC-like 1	-2.484	$4.82x10^{-03}$
201732_s_at	CLCN3	chloride channel 3	-1.963	$3.00x10^{-03}$
201735_s_at	CLCN3	chloride channel 3	-2.402	$7.82x10^{-03}$
242913_at	CLIC6	chloride intracellular channel 6	-3.413	$1.41x10^{-03}$
201768_s_at	CLINT1	clathrin interactor 1	-3.281	$2.28x10^{-05}$
211136_s_at	CLPTM1	cleft lip and palate associated transmembrane protein 1	-3.028	$2.88x10^{-03}$
1554677_s_at	CMTM4	CKLF-like MARVEL transmembrane domain containing 4	-2.094	$5.73x10^{-04}$
1562273_at	CNGA4	cyclic nucleotide gated channel alpha 4	-2.726	$9.45x10^{-03}$
1554052_at	CNOT1	CCR4-NOT transcription complex, subunit 1	-3.631	$4.56x10^{-04}$
1552496_a_at	COBL	cordon-bleu homolog (mouse)	-2.672	$6.97x10^{-03}$
1554339_a_at	COG3	component of oligomeric golgi complex 3	-3.319	$6.18x10^{-03}$
214336_s_at	COPA	coatomer protein complex, subunit alpha	-3.675	$3.56x10^{-06}$
213736_at	COX5B	Cytochrome c oxidase subunit Vb	2.066	$2.41x10^{-03}$
201942_s_at	CPD	carboxypeptidase D	-2.827	$4.51x10^{-04}$
233208_x_at	CPSF2	cleavage and polyadenylation specific factor 2, 100kDa	-2.097	$8.93x10^{-03}$
221673_s_at	CSNK1G1	casein kinase 1, gamma 1	-2.380	$2.41x10^{-03}$
212075_s_at	CSNK2A1	casein kinase 2, alpha 1 polypeptide	-2.505	$1.69x10^{-04}$
204459_at	CSTF2	cleavage stimulation factor, 3' pre-RNA, subunit 2, 64kDa	-1.575	$8.38x10^{-03}$
223271_s_at	CTDSPL2	CTD (carboxy-terminal domain, RNA polymerase II, polypeptide A) small phosphatase like 2	-1.969	$7.63x10^{-03}$
1558214_s_at	CTNNA1	catenin (cadherin-associated protein), alpha 1, 102kDa	-6.141	$6.54x10^{-04}$
1557944_s_at	CTNND1	catenin (cadherin-associated protein), delta 1	-3.683	$8.54x10^{-04}$
1555467_a_at	CUGBP1	CUG triplet repeat, RNA binding protein 1	-2.165	$6.27x10^{-03}$
1555716_a_at	CXADR	coxsackie virus and adenovirus receptor	-2.667	$3.55x10^{-03}$

Additional File 2. Significant Genes in the Small Airways Epithelium of Smokers with COPD Between Chips that Failed QC and Chips that Passed QC[1] (cont., page 6)

Probe set ID	Gene symbol	Gene title	Fold-change (Fail QC/Pass QC)	p value
230532_at	CXorf38	chromosome X open reading frame 38	-1.669	$6.93x10^{-03}$
1554574_a_at	CYB5R3	cytochrome b5 reductase 3	-3.408	$3.78x10^{-03}$
203890_s_at	DAPK3	death-associated protein kinase 3	-2.297	$9.69x10^{-03}$
212595_s_at	DAZAP2	DAZ associated protein 2	-3.174	$4.73x10^{-03}$
205369_x_at	DBT	dihydrolipoamide branched chain transacylase E2	-3.343	$2.80x10^{-03}$
200702_s_at	DDX24	DEAD (Asp-Glu-Ala-Asp) box polypeptide 24	-2.702	$8.21x10^{-04}$
1559954_s_at	DDX42	DEAD (Asp-Glu-Ala-Asp) box polypeptide 42	-4.920	$2.35x10^{-07}$
223662_x_at	DDX59	DEAD (Asp-Glu-Ala-Asp) box polypeptide 59	1.728	$3.79x10^{-03}$
1554352_s_at	DENND4A	DENN/MADD domain containing 4A	-1.684	$3.02x10^{-03}$
234728_s_at	DHX35	DEAH (Asp-Glu-Ala-His) box polypeptide 35	-3.547	$7.90x10^{-04}$
212105_s_at	DHX9	DEAH (Asp-Glu-Ala-His) box polypeptide 9	-3.995	$4.16x10^{-03}$
212107_s_at	DHX9	DEAH (Asp-Glu-Ala-His) box polypeptide 9	-4.174	$4.82x10^{-05}$
213514_s_at	DIAPH1	diaphanous homolog 1 (Drosophila)	-2.163	$7.63x10^{-03}$
1558342_x_at	DIXDC1	DIX domain containing 1	-2.902	$3.66x10^{-03}$
211150_s_at	DLAT	dihydrolipoamide S-acetyltransferase	-2.102	$6.35x10^{-03}$
202514_at	DLG1	discs, large homolog 1 (Drosophila)	-2.177	$9.04x10^{-03}$
233056_x_at	DLGAP4	discs, large (Drosophila) homolog-associated protein 4	1.886	$9.11x10^{-03}$
1562462_at	DNAH10	dynein, axonemal, heavy chain 10	-3.082	$2.48x10^{-05}$
1553159_at	DNAH11	dynein, axonemal, heavy chain 11	-2.609	$2.42x10^{-03}$
1563290_at	DNAH3	dynein, axonemal, heavy chain 3	-4.896	$5.27x10^{-03}$
220725_x_at	DNAH3	Dynein, axonemal, heavy chain 3	2.036	$8.19x10^{-03}$
1565337_at	DNAH6	dynein, axonemal, heavy chain 6	-4.729	$2.81x10^{-03}$
1552957_at	DNAH6 /// DNHL1 /// LOC200383	dynein, axonemal, heavy chain 6 /// dynein heavy chain-like 1 /// similar to Dynein heavy chain at 16F	-2.348	$8.71x10^{-03}$
234893_s_at	DNAH6 /// DNHL1 /// LOC200383	dynein, axonemal, heavy chain 6 /// dynein heavy chain-like 1 /// similar to Dynein heavy chain at 16F	-3.170	$8.84x10^{-04}$
221668_s_at	DNAI2	dynein, axonemal, intermediate chain 2	-3.104	$2.41x10^{-04}$
221781_s_at	DNAJC10	DnaJ (Hsp40) homolog, subfamily C, member 10	-2.433	$2.24x10^{-03}$
223446_s_at	DTNBP1	dystrobrevin binding protein 1	-1.946	$9.59x10^{-03}$
1553983_at	DTYMK	deoxythymidylate kinase (thymidylate kinase)	1.762	$9.71x10^{-04}$
1554648_a_at	DUOXA1	dual oxidase maturation factor 1	-2.088	$1.08x10^{-03}$
201044_x_at	DUSP1	dual specificity phosphatase 1	-2.756	$5.00x10^{-03}$
201537_s_at	DUSP3	dual specificity phosphatase 3	-2.666	$3.04x10^{-03}$
201538_s_at	DUSP3	dual specificity phosphatase 3	-6.163	$2.65x10^{-03}$
219207_at	EDC3	enhancer of mRNA decapping 3 homolog (S. cerevisiae)	-2.158	$7.70x10^{-03}$

Probe set ID	Gene symbol	Gene title	Fold-change (Fail QC/Pass QC)	p value
219850_s_at	EHF	ets homologous factor	-2.229	$9.78x10^{-03}$
222932_at	EHF	ets homologous factor	-3.221	$2.89x10^{-03}$
224189_x_at	EHF	ets homologous factor	-2.883	$1.56x10^{-04}$
208670_s_at	EID1	EP300 interacting inhibitor of differentiation 1	-2.373	$7.73x10^{-03}$
200596_s_at	EIF3A	eukaryotic translation initiation factor 3, subunit A	-2.233	$6.02x10^{-03}$
1555996_s_at	EIF4A2	eukaryotic translation initiation factor 4A, isoform 2	-3.382	$1.12x10^{-03}$
208624_s_at	EIF4G1	eukaryotic translation initiation factor 4 gamma, 1	-2.475	$2.61x10^{-03}$
212420_at	ELF1	E74-like factor 1 (ets domain transcription factor)	-2.983	$7.57x10^{-04}$
206127_at	ELK3	ELK3, ETS-domain protein (SRF accessory protein 2)	-2.488	$4.51x10^{-04}$
220386_s_at	EML4	echinoderm microtubule associated protein like 4	-2.438	$1.37x10^{-03}$
217294_s_at	ENO1	enolase 1, (alpha)	-4.314	$5.33x10^{-03}$
1555358_a_at	ENTPD4	ectonucleoside triphosphate diphosphohydrolase 4	-5.448	$1.63x10^{-03}$
220120_s_at	EPB41L4A	erythrocyte membrane protein band 4.1 like 4A	-3.203	$8.71x10^{-03}$
200842_s_at	EPRS	glutamyl-prolyl-tRNA synthetase	-2.749	$7.74x10^{-04}$
210930_s_at	ERBB2	v-erb-b2 erythroblastic leukemia viral oncogene homolog 2, neuro/glioblastoma derived oncogene homolog (avian)	-6.151	$6.69x10^{-03}$
202444_s_at	ERLIN1	ER lipid raft associated 1	-4.246	$2.60x10^{-04}$
210011_s_at	EWSR1	Ewing sarcoma breakpoint region 1	-2.004	$9.21x10^{-03}$
1555808_a_at	EXDL2	exonuclease 3'-5' domain-like 2	-6.009	$7.05x10^{-03}$
219349_s_at	EXOC2	exocyst complex component 2	-2.028	$8.75x10^{-03}$
240528_s_at	EXOC4	exocyst complex component 4	-3.345	$2.73x10^{-03}$
233924_s_at	EXOC6	exocyst complex component 6	-2.456	$3.78x10^{-03}$
212035_s_at	EXOC7	exocyst complex component 7	-1.902	$9.02x10^{-03}$
208621_s_at	EZR	ezrin	-6.072	$3.56x10^{-06}$
217234_s_at	EZR	ezrin	-3.542	$1.70x10^{-04}$
221664_s_at	F11R	F11 receptor	-3.009	$2.07x10^{-03}$
226697_at	FAM114A1	family with sequence similarity 114, member A1	-3.260	$3.61x10^{-04}$
1555945_s_at	FAM120A	family with sequence similarity 120A	-1.883	$3.93x10^{-03}$
200767_s_at	FAM120A	family with sequence similarity 120A	-2.533	$5.96x10^{-04}$
1569025_s_at	FAM13A1	family with sequence similarity 13, member A1	-2.655	$6.47x10^{-04}$
213896_x_at	FAM149B1	family with sequence similarity 149, member B1	-1.593	$8.77x10^{-03}$

Additional File 2. Significant Genes in the Small Airways Epithelium of Smokers with COPD Between Chips that Failed QC and Chips that Passed QC[1] (cont., page 8)

Probe set ID	Gene symbol	Gene title	Fold-change (Fail QC/Pass QC)	p value
222158_s_at	FAM152A	family with sequence similarity 152, member A	-2.427	$4.69x10^{-03}$
1557385_at	FAM161A	family with sequence similarity 161, member A	-5.274	$9.01x10^{-03}$
212835_at	FAM175B	family with sequence similarity 175, member B	-1.718	$3.72x10^{-03}$
223038_s_at	FAM60A	family with sequence similarity 60, member A	-2.315	$3.81x10^{-03}$
1555829_at	FAM62B	family with sequence similarity 62 (C2 domain containing) member B	-4.517	$3.86x10^{-03}$
1555830_s_at	FAM62B	family with sequence similarity 62 (C2 domain containing) member B	-3.738	$2.39x10^{-04}$
215719_x_at	FAS	Fas (TNF receptor superfamily, member 6)	-2.088	$3.20x10^{-03}$
216252_x_at	FAS	Fas (TNF receptor superfamily, member 6)	-2.287	$8.23x10^{-03}$
219608_s_at	FBXO38	F-box protein 38	-7.264	$2.95x10^{-03}$
225099_at	FBXO45	F-box protein 45	-1.526	$7.69x10^{-03}$
1566509_s_at	FBXO9	F-box protein 9	-1.901	$2.31x10^{-03}$
215404_x_at	FGFR1	fibroblast growth factor receptor 1	2.307	$7.46x10^{-03}$
1570515_a_at	FILIP1	filamin A interacting protein 1	-5.605	$4.93x10^{-03}$
1564160_at	FLJ16686	FLJ16686 protein	-4.353	$2.75x10^{-03}$
219858_s_at	FLJ20160	FLJ20160 protein	-3.518	$7.83x10^{-04}$
230047_at	FLJ32810	hypothetical protein FLJ32810	-1.780	$4.69x10^{-03}$
1564193_at	FLJ39061	hypothetical protein FLJ39061	-2.723	$2.60x10^{-04}$
215300_s_at	FMO5	flavin containing monooxygenase 5	-1.914	$4.95x10^{-03}$
215910_s_at	FNDC3A	fibronectin type III domain containing 3A	-3.670	$6.97x10^{-03}$
1773_at	FNTB	farnesyltransferase, CAAX box, beta	-2.170	$1.56x10^{-03}$
223985_at	FSD1L	fibronectin type III and SPRY domain containing 1-like	-2.038	$3.81x10^{-03}$
212380_at	FTSJD2	FtsJ methyltransferase domain containing 2	-1.829	$5.33x10^{-03}$
217370_x_at	FUS	fusion (involved in t(12;16) in malignant liposarcoma)	-3.574	$2.09x10^{-03}$
210178_x_at	FUSIP1 /// LOC642558	FUS interacting protein (serine/arginine-rich) 1 /// similar to FUS interacting protein (serine-arginine rich) 1	-3.410	$6.93x10^{-04}$
221187_s_at	FUZ	fuzzy homolog (Drosophila)	-2.310	$5.89x10^{-03}$
224642_at	FYTTD1	forty-two-three domain containing 1	-3.006	$5.77x10^{-04}$
211810_s_at	GALC	galactosylceramidase	-2.915	$1.19x10^{-03}$
205219_s_at	GALK2	galactokinase 2	-2.669	$5.59x10^{-03}$
1568618_a_at	GALNT1	UDP-N-acetyl-alpha-D-galactosamine:polypeptide N-acetylgalactosaminyltransferase 1 (GalNAc-T1)	-3.062	$2.03x10^{-03}$
201723_s_at	GALNT1	UDP-N-acetyl-alpha-D-galactosamine:polypeptide N-acetylgalactosaminyltransferase 1 (GalNAc-	-1.549	$6.04x10^{-03}$

Additional File 2. Significant Genes in the Small Airways Epithelium of Smokers with COPD Between Chips that Failed QC and Chips that Passed QC[1] (cont., page 9)

Probe set ID	Gene symbol	Gene title	Fold-change (Fail QC/Pass QC)	p value
		T1)		
229555_at	GALNT5	UDP-N-acetyl-alpha-D-galactosamine:polypeptide N-acetylgalactosaminyltransferase 5 (GalNAc-T5)	-1.929	$8.96x10^{-03}$
214869_x_at	GAPVD1	GTPase activating protein and VPS9 domains 1	-2.176	$6.73x10^{-05}$
201439_at	GBF1	golgi-specific brefeldin A resistance factor 1	-1.953	$7.82x10^{-03}$
239761_at	GCNT1	glucosaminyl (N-acetyl) transferase 1, core 2 (beta-1,6-N-acetylglucosaminyltransferase)	-1.901	$3.72x10^{-03}$
211020_at	GCNT2	glucosaminyl (N-acetyl) transferase 2, I-branching enzyme (I blood group)	-6.874	$3.15x10^{-03}$
221279_at	GDAP1	ganglioside-induced differentiation-associated protein 1	-5.068	$2.93x10^{-03}$
202593_s_at	GDE1	glycerophosphodiester phosphodiesterase 1	-2.505	$7.91x10^{-03}$
232296_s_at	GFM1	G elongation factor, mitochondrial 1	-2.927	$7.31x10^{-05}$
231917_at	GFM2	G elongation factor, mitochondrial 2	-1.950	$1.19x10^{-03}$
231918_s_at	GFM2	G elongation factor, mitochondrial 2	-3.609	$1.48x10^{-04}$
202721_s_at	GFPT1	glutamine-fructose-6-phosphate transaminase 1	-3.511	$8.08x10^{-03}$
214190_x_at	GGA2	golgi associated, gamma adaptin ear containing, ARF binding protein 2	-5.982	$8.57x10^{-03}$
209411_s_at	GGA3	golgi associated, gamma adaptin ear containing, ARF binding protein 3	-1.585	$4.41x10^{-03}$
207387_s_at	GK	glycerol kinase	-2.073	$1.86x10^{-03}$
1560316_s_at	GLCCI1	glucocorticoid induced transcript 1	-2.149	$5.68x10^{-03}$
214730_s_at	GLG1	golgi apparatus protein 1	-2.113	$4.96x10^{-03}$
230258_at	GLIS3	GLIS family zinc finger 3	-2.184	$3.73x10^{-03}$
217202_s_at	GLUL	glutamate-ammonia ligase (glutamine synthetase)	-3.045	$6.97x10^{-03}$
206917_at	GNA13	guanine nucleotide binding protein (G protein), alpha 13	-3.852	$6.77x10^{-03}$
203676_at	GNS	glucosamine (N-acetyl)-6-sulfatase	-2.394	$3.67x10^{-03}$
204384_at	GOLGA2	golgi autoantigen, golgin subfamily a, 2	-14.862	$3.07x10^{-05}$
238689_at	GPR110	G protein-coupled receptor 110	-2.677	$2.11x10^{-03}$
228950_s_at	GPR177	G protein-coupled receptor 177	-2.303	$9.76x10^{-03}$
1552685_a_at	GRHL1	grainyhead-like 1 (Drosophila)	-2.056	$2.10x10^{-03}$
217008_s_at	GRM7	glutamate receptor, metabotropic 7	-2.415	$1.15x10^{-03}$
202453_s_at	GTF2H1	general transcription factor IIH, polypeptide 1, 62kDa	-1.871	$8.77x10^{-03}$
223758_s_at	GTF2H2	general transcription factor IIH, polypeptide 2, 44kDa	-3.075	$1.38x10^{-03}$
210892_s_at	GTF2I	general transcription factor II, i	-2.935	$7.36x10^{-05}$
215913_s_at	GULP1	GULP, engulfment adaptor PTB domain	-2.146	$7.46x10^{-03}$

Additional File 2. Significant Genes in the Small Airways Epithelium of Smokers with COPD Between Chips that Failed QC and Chips that Passed QC[1] (cont., page 10)

Probe set ID	Gene symbol	Gene title	Fold-change (Fail QC/Pass QC)	p value
		containing 1		
214501_s_at	H2AFY	H2A histone family, member Y	-2.034	$2.26x10^{-03}$
208629_s_at	HADHA	hydroxyacyl-Coenzyme A dehydrogenase/3-ketoacyl-Coenzyme A thiolase/enoyl-Coenzyme A hydratase (trifunctional protein), alpha subunit	-3.209	$7.63x10^{-05}$
219484_at	HCFC2	host cell factor C2	-1.740	$3.62x10^{-03}$
216591_s_at	hCG_1776980 /// SDHC	hCG1776980 /// succinate dehydrogenase complex, subunit C, integral membrane protein, 15kDa	-2.708	$1.08x10^{-03}$
1552516_a_at	HIPK1	homeodomain interacting protein kinase 1	-6.203	$4.51x10^{-04}$
200679_x_at	HMGB1	high-mobility group box 1	-2.635	$1.26x10^{-03}$
200751_s_at	HNRNPC	heterogeneous nuclear ribonucleoprotein C (C1/C2)	-2.679	$1.08x10^{-03}$
208765_s_at	HNRNPR	heterogeneous nuclear ribonucleoprotein R	-2.951	$2.39x10^{-04}$
1554678_s_at	HNRPDL	heterogeneous nuclear ribonucleoprotein D-like	-2.303	$5.29x10^{-03}$
211220_s_at	HSF2	heat shock transcription factor 2	-2.240	$9.69x10^{-03}$
211968_s_at	HSP90AA1	heat shock protein 90kDa alpha (cytosolic), class A member 1	-1.758	$2.28x10^{-03}$
1557910_at	HSP90AB1	heat shock protein 90kDa alpha (cytosolic), class B member 1	-7.064	$1.31x10^{-04}$
200598_s_at	HSP90B1	heat shock protein 90kDa beta (Grp94), member 1	-3.637	$2.28x10^{-05}$
211016_x_at	HSPA4	heat shock 70kDa protein 4	-3.448	$1.89x10^{-04}$
200806_s_at	HSPD1	heat shock 60kDa protein 1 (chaperonin)	-3.262	$1.10x10^{-04}$
208744_x_at	HSPH1	heat shock 105kDa/110kDa protein 1	-4.565	$4.45x10^{-04}$
1554205_s_at	ICA1L	islet cell autoantigen 1,69kDa-like	-2.076	$2.60x10^{-04}$
223881_at	ICA1L	islet cell autoantigen 1,69kDa-like	-2.249	$9.23x10^{-03}$
203328_x_at	IDE	insulin-degrading enzyme	-2.067	$7.50x10^{-03}$
217496_s_at	IDE	insulin-degrading enzyme	-2.197	$8.04x10^{-03}$
202438_x_at	IDS	iduronate 2-sulfatase	-2.300	$5.90x10^{-03}$
1552865_a_at	IFLTD1	intermediate filament tail domain containing 1	-2.384	$1.54x10^{-03}$
222519_s_at	IFT57	intraflagellar transport 57 homolog (Chlamydomonas)	-3.212	$7.10x10^{-04}$
201163_s_at	IGFBP7	insulin-like growth factor binding protein 7	1.782	$7.15x10^{-03}$
211027_s_at	IKBKB	inhibitor of kappa light polypeptide gene enhancer in B-cells, kinase beta	-4.866	$7.63x10^{-03}$
210904_s_at	IL13RA1	interleukin 13 receptor, alpha 1	-3.152	$1.99x10^{-04}$
211612_s_at	IL13RA1	interleukin 13 receptor, alpha 1	-2.307	$9.49x10^{-03}$
211000_s_at	IL6ST	interleukin 6 signal transducer (gp130, oncostatin M receptor)	-3.000	$2.60x10^{-03}$
208930_s_at	ILF3	interleukin enhancer binding factor 3, 90kDa	-4.427	$6.47x10^{-04}$
218516_s_at	IMPAD1	inositol monophosphatase domain containing	-1.916	$7.85x10^{-03}$

Additional File 2. Significant Genes in the Small Airways Epithelium of Smokers with COPD Between Chips that Failed QC and Chips that Passed QC[1] (cont., page 11)

Probe set ID	Gene symbol	Gene title	Fold-change (Fail QC/Pass QC)	p value
		1		
65133_i_at	INO80B /// WBP1	INO80 complex subunit B /// WW domain binding protein 1	-2.835	$3.57x10^{-03}$
1554740_a_at	IPP	intracisternal A particle-promoted polypeptide	-3.030	$2.48x10^{-05}$
215130_s_at	IQCK	IQ motif containing K	-2.729	$2.61x10^{-03}$
210840_s_at	IQGAP1	IQ motif containing GTPase activating protein 1	-1.833	$9.59x10^{-04}$
232349_x_at	IQWD1	IQ motif and WD repeats 1	-1.856	$3.06x10^{-03}$
1553530_a_at	ITGB1	integrin, beta 1 (fibronectin receptor, beta polypeptide, antigen CD29 includes MDF2, MSK12)	-2.421	$2.06x10^{-03}$
211323_s_at	ITPR1	inositol 1,4,5-triphosphate receptor, type 1	-2.798	$2.61x10^{-03}$
1552610_a_at	JAK1	Janus kinase 1 (a protein tyrosine kinase)	-3.459	$6.93x10^{-03}$
205842_s_at	JAK2	Janus kinase 2 (a protein tyrosine kinase)	-2.808	$6.19x10^{-03}$
214861_at	JMJD2C	jumonji domain containing 2C	2.338	$1.23x10^{-03}$
223584_s_at	KBTBD2	kelch repeat and BTB (POZ) domain containing 2	-2.070	$1.18x10^{-03}$
223585_x_at	KBTBD2	kelch repeat and BTB (POZ) domain containing 2	-4.959	$5.65x10^{-03}$
223765_s_at	KBTBD4	kelch repeat and BTB (POZ) domain containing 4	-2.966	$5.64x10^{-03}$
208514_at	KCNE1	potassium voltage-gated channel, Isk-related family, member 1	-3.697	$2.60x10^{-04}$
204678_s_at	KCNK1	potassium channel, subfamily K, member 1	-2.308	$3.40x10^{-04}$
201729_s_at	KIAA0100	KIAA0100	-2.002	$8.34x10^{-03}$
200616_s_at	KIAA0152	KIAA0152	-2.634	$1.86x10^{-03}$
201777_s_at	KIAA0494	KIAA0494	-2.475	$1.73x10^{-03}$
204074_s_at	KIAA0562	KIAA0562	-2.179	$1.67x10^{-03}$
207305_s_at	KIAA1012	KIAA1012	-1.600	$3.55x10^{-03}$
212794_s_at	KIAA1033	KIAA1033	-3.281	$6.16x10^{-03}$
1554132_a_at	KIAA1128	KIAA1128	-2.424	$6.22x10^{-03}$
201104_x_at	KIAA1245 /// NBPF1 /// NBPF10 /// NBPF11 /// NBPF12 /// NBPF14 /// NBPF15 /// NBPF16 /// NBPF20 /// NBPF8 /// NBPF9 /// XXyac-YX155B6.1	KIAA1245 /// neuroblastoma breakpoint family, member 1 /// neuroblastoma breakpoint family, member 10 /// neuroblastoma breakpoint family, member 11 /// neuroblastoma breakpoint family, member 12 /// neuroblastoma breakpoint family, member 14 /// neuroblastoma breakpoint family, member 15 /// neuroblastoma breakpoint family, member 16 /// neuroblastoma breakpoint family, member 20 /// neuroblastoma breakpoint family, member 8 /// neuroblastoma breakpoint family, member 9 /// CLIP-190-like	-2.332	$4.97x10^{-05}$

Additional File 2. Significant Genes in the Small Airways Epithelium of Smokers with COPD Between Chips that Failed QC and Chips that Passed QC[1] (cont., page 12)

Probe set ID	Gene symbol	Gene title	Fold-change (Fail QC/Pass QC)	p value
214693_x_at	KIAA1245 /// NBPF1 /// NBPF10 /// NBPF11 /// NBPF14 /// NBPF15 /// NBPF16 /// NBPF20 /// NBPF8 /// NBPF9 /// XXyac-YX155B6.1	KIAA1245 /// neuroblastoma breakpoint family, member 1 /// neuroblastoma breakpoint family, member 10 /// neuroblastoma breakpoint family, member 11 /// neuroblastoma breakpoint family, member 14 /// neuroblastoma breakpoint family, member 15 /// neuroblastoma breakpoint family, member 16 /// neuroblastoma breakpoint family, member 20 /// neuroblastoma breakpoint family, member 8 /// neuroblastoma breakpoint family, member 9 /// CLIP-190-like	-6.430	$1.04x10^{-05}$
215434_x_at	KIAA1245 /// NBPF1 /// NBPF10 /// NBPF11 /// NBPF14 /// NBPF15 /// NBPF16 /// NBPF20 /// NBPF8 /// NBPF9 /// XXyac-YX155B6.1	KIAA1245 /// neuroblastoma breakpoint family, member 1 /// neuroblastoma breakpoint family, member 10 /// neuroblastoma breakpoint family, member 11 /// neuroblastoma breakpoint family, member 14 /// neuroblastoma breakpoint family, member 15 /// neuroblastoma breakpoint family, member 16 /// neuroblastoma breakpoint family, member 20 /// neuroblastoma breakpoint family, member 8 /// neuroblastoma breakpoint family, member 9 /// CLIP-190-like	-2.943	$2.60x10^{-03}$
243349_at	KIAA1324	KIAA1324	-4.326	$8.62x10^{-03}$
223254_s_at	KIAA1333	KIAA1333	-2.873	$2.62x10^{-03}$
225508_at	KIAA1468	KIAA1468	-1.810	$5.27x10^{-03}$
238490_at	KIAA2026	KIAA2026	-2.393	$3.78x10^{-03}$
212162_at	KIDINS220	kinase D-interacting substrate of 220 kDa	-3.203	$2.51x10^{-03}$
201992_s_at	KIF5B	kinesin family member 5B	-2.121	$9.45x10^{-03}$
212878_s_at	KLC1	kinesin light chain 1	-2.295	$6.19x10^{-03}$
219657_s_at	KLF3	Kruppel-like factor 3 (basic)	-2.215	$7.20x10^{-03}$
225140_at	KLF3	Kruppel-like factor 3 (basic)	-2.288	$1.62x10^{-04}$
209254_at	KLHDC10	kelch domain containing 10	-3.404	$8.79x10^{-04}$
221986_s_at	KLHL24	kelch-like 24 (Drosophila)	-2.373	$9.81x10^{-04}$
1555275_a_at	KLHL6	kelch-like 6 (Drosophila)	-2.124	$4.05x10^{-03}$
1560397_s_at	KLHL6	kelch-like 6 (Drosophila)	-3.162	$8.04x10^{-03}$
208974_x_at	KPNB1	karyopherin (importin) beta 1	-2.326	$9.32x10^{-03}$
222585_x_at	KRCC1	lysine-rich coiled-coil 1	-2.196	$9.23x10^{-03}$
1555815_a_at	L3MBTL2	l(3)mbt-like 2 (Drosophila)	-2.412	$1.19x10^{-03}$
202019_s_at	LANCL1	LanC lantibiotic synthetase component C-like 1 (bacterial)	-1.948	$4.66x10^{-03}$
238959_at	LARP4	La ribonucleoprotein domain family, member 4	-5.550	$1.86x10^{-03}$

Additional File 2. Significant Genes in the Small Airways Epithelium of Smokers with COPD Between Chips that Failed QC and Chips that Passed QC[1] (cont., page 13)

Probe set ID	Gene symbol	Gene title	Fold-change (Fail QC/Pass QC)	p value
242019_at	LASS6	LAG1 homolog, ceramide synthase 6	-3.114	$5.16x10^{-04}$
206235_at	LIG4	ligase IV, DNA, ATP-dependent	-2.636	$3.81x10^{-03}$
222456_s_at	LIMA1	LIM domain and actin binding 1	-4.417	$2.55x10^{-03}$
222457_s_at	LIMA1	LIM domain and actin binding 1	-4.008	$2.34x10^{-05}$
217475_s_at	LIMK2	LIM domain kinase 2	-3.198	$3.00x10^{-03}$
207198_s_at	LIMS1	LIM and senescent cell antigen-like domains 1	-3.511	$3.09x10^{-04}$
235871_at	LIPH	lipase, member H	-2.162	$1.35x10^{-04}$
203294_s_at	LMAN1	lectin, mannose-binding, 1	-5.214	$1.79x10^{-03}$
1553284_s_at	LMLN	leishmanolysin-like (metallopeptidase M8 family)	-2.794	$4.79x10^{-04}$
212806_at	LOC100129762 /// PRUNE2	similar to KIAA0367 /// prune homolog 2 (Drosophila)	-3.053	$3.91x10^{-04}$
207247_s_at	LOC100130829 /// ZFX /// ZFY	hypothetical protein LOC100130829 /// zinc finger protein, X-linked /// zinc finger protein, Y-linked	-2.844	$2.40x10^{-03}$
222406_s_at	LOC100132235 /// PNRC2	hypothetical protein LOC100132235 /// proline-rich nuclear receptor coactivator 2	-3.921	$9.23x10^{-04}$
231247_s_at	LOC100132999	hypothetical protein LOC100132999	2.609	$3.93x10^{-03}$
201383_s_at	LOC100133166 /// NBR1	similar to neighbor of BRCA1 gene 1 /// neighbor of BRCA1 gene 1	-3.045	$2.62x10^{-03}$
220219_s_at	LOC100133503 /// LOC644397 /// LRRC37A /// LRRC37A2 /// LRRC37A3 /// LRRC37A4	similar to LRRC37A3 protein /// similar to hCG2036502 /// leucine rich repeat containing 37A /// leucine rich repeat containing 37, member A2 /// leucine rich repeat containing 37, member A3 /// leucine rich repeat containing 37, member A4 (pseudogene)	-2.648	$8.37x10^{-03}$
1552716_at	LOC100134025 /// SPEF2	hypothetical protein LOC100134025 /// sperm flagellar 2	-2.641	$5.82x10^{-03}$
225920_at	LOC148413	hypothetical LOC148413	-2.411	$9.80x10^{-03}$
215978_x_at	LOC152719	hypothetical protein LOC152719	2.435	$3.81x10^{-03}$
239466_at	LOC344595	hypothetical LOC344595	-2.252	$2.60x10^{-03}$
224605_at	LOC401152	HCV F-transactivated protein 1	-2.682	$5.53x10^{-03}$
214107_x_at	LOC440434	hypothetical protein FLJ11822	-2.093	$2.77x10^{-05}$
215090_x_at	LOC440434	hypothetical protein FLJ11822	-1.824	$3.49x10^{-03}$
212392_s_at	LOC652526 /// PDE4DIP	similar to phosphodiesterase 4D interacting protein isoform 2 /// phosphodiesterase 4D interacting protein	-5.262	$4.93x10^{-03}$
204860_s_at	LOC652755 /// NAIP	similar to Baculoviral IAP repeat-containing protein 1 (Neuronal apoptosis inhibitory protein) /// NLR family, apoptosis inhibitory protein	-4.027	$9.20x10^{-03}$
243124_at	LOC653390	RRN3 RNA polymerase I transcription factor homolog (S. cerevisiae) pseudogene	-2.850	$4.70x10^{-03}$

Additional File 2. Significant Genes in the Small Airways Epithelium of Smokers with COPD Between Chips that Failed QC and Chips that Passed QC[1] (cont., page 14)

Probe set ID	Gene symbol	Gene title	Fold-change (Fail QC/Pass QC)	p value
221618_s_at	LOC728198 /// TAF9B	similar to transcription associated factor TAFII31L /// TAF9B RNA polymerase II, TATA box binding protein (TBP)-associated factor, 31kDa	-3.313	$5.09x10^{-03}$
215667_x_at	LOC730324	similar to postmeiotic segregation increased 2-like 2	-1.859	$6.96x10^{-03}$
220816_at	LPAR3	lysophosphatidic acid receptor 3	-2.927	$2.85x10^{-03}$
202459_s_at	LPIN2	lipin 2	-2.162	$1.26x10^{-03}$
202821_s_at	LPP	LIM domain containing preferred translocation partner in lipoma	-3.067	$4.17x10^{-03}$
223200_s_at	LSG1	large subunit GTPase 1 homolog (S. cerevisiae)	-2.785	$2.18x10^{-03}$
200900_s_at	M6PR	mannose-6-phosphate receptor (cation dependent)	-4.042	$7.36x10^{-05}$
214894_x_at	MACF1	microtubule-actin crosslinking factor 1	-2.092	$7.63x10^{-03}$
215222_x_at	MACF1	microtubule-actin crosslinking factor 1	-2.738	$2.07x10^{-03}$
222944_s_at	MAGIX	MAGI family member, X-linked	-3.757	$6.04x10^{-03}$
224568_x_at	MALAT1	metastasis associated lung adenocarcinoma transcript 1 (non-protein coding)	-7.583	$4.85x10^{-03}$
235106_at	MAML2	mastermind-like 2 (Drosophila)	-1.911	$3.78x10^{-03}$
214577_at	MAP1B	microtubule-associated protein 1B	-4.579	$9.63x10^{-03}$
203265_s_at	MAP2K4	mitogen-activated protein kinase kinase 4	-2.160	$3.38x10^{-03}$
211083_s_at	MAP3K13	mitogen-activated protein kinase kinase kinase 13	-8.220	$1.08x10^{-03}$
221695_s_at	MAP3K2	mitogen-activated protein kinase kinase kinase 2	-2.870	$2.48x10^{-03}$
211536_x_at	MAP3K7	mitogen-activated protein kinase kinase kinase 7	-2.099	$5.01x10^{-03}$
211537_x_at	MAP3K7	mitogen-activated protein kinase kinase kinase 7	-1.850	$8.33x10^{-03}$
210284_s_at	MAP3K7IP2	mitogen-activated protein kinase kinase kinase 7 interacting protein 2	-2.309	$3.23x10^{-03}$
203553_s_at	MAP4K5	mitogen-activated protein kinase kinase kinase kinase 5	-1.895	$9.06x10^{-03}$
235672_at	MAP6	microtubule-associated protein 6	-2.437	$5.73x10^{-04}$
215471_s_at	MAP7	microtubule-associated protein 7	-2.499	$7.70x10^{-03}$
239415_at	MAP9	microtubule-associated protein 9	-4.069	$6.04x10^{-03}$
208351_s_at	MAPK1	mitogen-activated protein kinase 1	-2.472	$2.23x10^{-03}$
211561_x_at	MAPK14	mitogen-activated protein kinase 14	-2.334	$1.00x10^{-03}$
229846_s_at	MAPKAP1	mitogen-activated protein kinase associated protein 1	-4.054	$4.66x10^{-03}$
203841_x_at	MAPRE3	microtubule-associated protein, RP/EB family, member 3	-8.349	$2.34x10^{-03}$
214270_s_at	MAPRE3	microtubule-associated protein, RP/EB	-3.153	$9.50x10^{-03}$

Additional File 2. Significant Genes in the Small Airways Epithelium of Smokers with COPD Between Chips that Failed QC and Chips that Passed QC[1] (cont., page 15)

Probe set ID	Gene symbol	Gene title	Fold-change (Fail QC/Pass QC)	p value
		family, member 3		
1558093_s_at	MATR3	matrin 3	-4.689	$1.24x10^{-04}$
205017_s_at	MBNL2	muscleblind-like 2 (Drosophila)	-3.808	$2.60x10^{-04}$
205018_s_at	MBNL2	muscleblind-like 2 (Drosophila)	-3.500	$2.06x10^{-03}$
212246_at	MCFD2	multiple coagulation factor deficiency 2	-1.640	$5.27x10^{-03}$
200796_s_at	MCL1	myeloid cell leukemia sequence 1 (BCL2-related)	-7.260	$2.76x10^{-03}$
226293_at	MED19	mediator complex subunit 19	-1.699	$2.18x10^{-03}$
223947_s_at	MED23	mediator complex subunit 23	-2.103	$9.81x10^{-04}$
211599_x_at	MET	met proto-oncogene (hepatocyte growth factor receptor)	-3.112	$2.60x10^{-04}$
213807_x_at	MET	met proto-oncogene (hepatocyte growth factor receptor)	-2.889	$8.41x10^{-04}$
213816_s_at	MET	met proto-oncogene (hepatocyte growth factor receptor)	-2.433	$9.43x10^{-03}$
213899_at	METAP2	methionyl aminopeptidase 2	-1.731	$9.45x10^{-03}$
216205_s_at	MFN2	mitofusin 2	-2.617	$2.91x10^{-04}$
242520_s_at	MGC33556	hypothetical LOC339541	-3.234	$6.93x10^{-04}$
1569057_s_at	MIA3	melanoma inhibitory activity family, member 3	-2.619	$5.33x10^{-03}$
213189_at	MINA	MYC induced nuclear antigen	-2.449	$3.20x10^{-04}$
1555820_a_at	MKS1	Meckel syndrome, type 1	-2.058	$9.45x10^{-03}$
212076_at	MLL	myeloid/lymphoid or mixed-lineage leukemia (trithorax homolog, Drosophila)	-2.287	$9.88x10^{-03}$
205408_at	MLLT10	myeloid/lymphoid or mixed-lineage leukemia (trithorax homolog, Drosophila); translocated to, 10	-2.028	$1.28x10^{-03}$
237211_x_at	MORN3	MORN repeat containing 3	-2.046	$8.72x10^{-03}$
219321_at	MPP5	membrane protein, palmitoylated 5 (MAGUK p55 subfamily member 5)	-2.580	$1.73x10^{-04}$
224247_s_at	MRPS10	mitochondrial ribosomal protein S10	-2.261	$1.12x10^{-03}$
212604_at	MRPS31	mitochondrial ribosomal protein S31	-1.998	$2.78x10^{-03}$
203207_s_at	MTFR1	mitochondrial fission regulator 1	-1.826	$9.86x10^{-03}$
214975_s_at	MTMR1	myotubularin related protein 1	-2.961	$2.42x10^{-03}$
224430_s_at	MTO1	mitochondrial translation optimization 1 homolog (S. cerevisiae)	-1.971	$5.94x10^{-03}$
212093_s_at	MTUS1	mitochondrial tumor suppressor 1	-3.398	$5.03x10^{-04}$
212095_s_at	MTUS1	mitochondrial tumor suppressor 1	-3.110	$1.19x10^{-03}$
227241_at	MUC15	mucin 15, cell surface associated	-3.308	$4.17x10^{-03}$
215649_s_at	MVK	mevalonate kinase	-6.058	$2.10x10^{-03}$
210480_s_at	MYO6	myosin VI	-2.479	$1.67x10^{-03}$
211139_s_at	NAB1	NGFI-A binding protein 1 (EGR1 binding protein 1)	-2.134	$9.09x10^{-03}$
1560339_s_at	NAP1L4	nucleosome assembly protein 1-like 4	-2.515	$3.96x10^{-03}$

Additional File 2. Significant Genes in the Small Airways Epithelium of Smokers with COPD Between Chips that Failed QC and Chips that Passed QC[1] (cont., page 16)

Probe set ID	Gene symbol	Gene title	Fold-change (Fail QC/Pass QC)	p value
201414_s_at	NAP1L4	nucleosome assembly protein 1-like 4	-1.751	$5.55x10^{-03}$
210048_at	NAPG	N-ethylmaleimide-sensitive factor attachment protein, gamma	-1.744	$9.49x10^{-03}$
219158_s_at	NARG1	NMDA receptor regulated 1	-3.017	$4.05x10^{-03}$
1555450_a_at	NARG1L	NMDA receptor regulated 1-like	-3.548	$9.91x10^{-03}$
218734_at	NAT11	N-acetyltransferase 11	-2.101	$7.25x10^{-03}$
205732_s_at	NCOA2	nuclear receptor coactivator 2	-2.164	$8.62x10^{-03}$
209060_x_at	NCOA3	nuclear receptor coactivator 3	-3.507	$1.88x10^{-04}$
211352_s_at	NCOA3	nuclear receptor coactivator 3	-3.687	$4.91x10^{-03}$
1553588_at	ND3 /// SH3KBP1	NADH dehydrogenase, subunit 3 (complex I) /// SH3-domain kinase binding protein 1	1.529	$3.94x10^{-03}$
222625_s_at	NDE1	nudE nuclear distribution gene E homolog 1 (A. nidulans)	-1.676	$9.89x10^{-03}$
224799_at	NDFIP2	Nedd4 family interacting protein 2	-1.915	$6.13x10^{-03}$
214279_s_at	NDRG2	NDRG family member 2	-2.812	$4.73x10^{-03}$
232146_at	NDUFC1	NADH dehydrogenase (ubiquinone) 1, subcomplex unknown, 1, 6kDa	-2.116	$6.19x10^{-03}$
207279_s_at	NEBL	nebulette	-1.730	$9.45x10^{-03}$
202150_s_at	NEDD9	neural precursor cell expressed, developmentally down-regulated 9	-2.599	$7.25x10^{-03}$
237227_at	NEK10	NIMA (never in mitosis gene a)- related kinase 10	-2.303	$6.44x10^{-03}$
1555082_a_at	NEK11	NIMA (never in mitosis gene a)- related kinase 11	-2.353	$1.64x10^{-04}$
211089_s_at	NEK3	NIMA (never in mitosis gene a)-related kinase 3	-2.891	$9.31x10^{-03}$
1557170_at	NEK8	NIMA (never in mitosis gene a)- related kinase 8	1.732	$4.51x10^{-03}$
1557172_x_at	NEK8	NIMA (never in mitosis gene a)- related kinase 8	1.705	$3.84x10^{-03}$
212808_at	NFATC2IP	nuclear factor of activated T-cells, cytoplasmic, calcineurin-dependent 2 interacting protein	-1.745	$5.89x10^{-03}$
1567013_at	NFE2L2	nuclear factor (erythroid-derived 2)-like 2	-3.430	$1.41x10^{-03}$
1553348_a_at	NFX1	nuclear transcription factor, X-box binding 1	-2.071	$3.04x10^{-03}$
207108_s_at	NIPBL	Nipped-B homolog (Drosophila)	-2.777	$1.80x10^{-03}$
234762_x_at	NLN	Neurolysin (metallopeptidase M3 family)	2.346	$2.31x10^{-03}$
218036_x_at	NMD3	NMD3 homolog (S. cerevisiae)	-2.265	$2.23x10^{-03}$
202784_s_at	NNT	nicotinamide nucleotide transhydrogenase	-1.934	$3.57x10^{-03}$
1554082_a_at	NOL9	nucleolar protein 9	-1.661	$7.40x10^{-03}$
227161_at	NOM1	nucleolar protein with MIF4G domain 1	-1.538	$6.16x10^{-03}$
210756_s_at	NOTCH2	Notch homolog 2 (Drosophila)	-2.639	$2.28x10^{-03}$
221628_s_at	N-PAC	cytokine-like nuclear factor n-pac	-7.285	$1.67x10^{-03}$
220128_s_at	NPAL2	NIPA-like domain containing 2	-2.461	$2.41x10^{-03}$

Additional File 2. Significant Genes in the Small Airways Epithelium of Smokers with COPD Between Chips that Failed QC and Chips that Passed QC[1] (cont., page 17)

Probe set ID	Gene symbol	Gene title	Fold-change (Fail QC/Pass QC)	p value
201454_s_at	NPEPPS	aminopeptidase puromycin sensitive	-2.561	$2.60x10^{-04}$
238844_s_at	NPHP1	nephronophthisis 1 (juvenile)	-2.687	$1.65x10^{-05}$
207978_s_at	NR4A3	nuclear receptor subfamily 4, group A, member 3	-4.868	$5.29x10^{-03}$
211844_s_at	NRP2	neuropilin 2	-5.648	$7.01x10^{-03}$
214632_at	NRP2	neuropilin 2	-1.941	$3.23x10^{-03}$
229225_at	NRP2	neuropilin 2	-1.884	$8.37x10^{-03}$
223178_s_at	NT5DC1	5'-nucleotidase domain containing 1	-1.648	$7.27x10^{-03}$
206303_s_at	NUDT4 /// NUDT4P1	nudix (nucleoside diphosphate linked moiety X)-type motif 4 /// nudix (nucleoside diphosphate linked moiety X)-type motif 4 pseudogene 1	-1.798	$9.50x10^{-03}$
218295_s_at	NUP50	nucleoporin 50kDa	-1.885	$7.46x10^{-03}$
209629_s_at	NXT2	nuclear transport factor 2-like export factor 2	-3.274	$3.96x10^{-03}$
239748_x_at	OCIAD1	OCIA domain containing 1	2.098	$4.09x10^{-03}$
208316_s_at	OCRL	oculocerebrorenal syndrome of Lowe	-2.471	$1.97x10^{-03}$
230926_s_at	ODF2L	outer dense fiber of sperm tails 2-like	-2.755	$6.44x10^{-03}$
1554152_a_at	OGDH	oxoglutarate (alpha-ketoglutarate) dehydrogenase (lipoamide)	-4.598	$9.76x10^{-03}$
209485_s_at	OSBPL1A	oxysterol binding protein-like 1A	-2.072	$2.23x10^{-03}$
223879_s_at	OXR1	oxidation resistance 1	-2.915	$3.11x10^{-03}$
1564494_s_at	P4HB	procollagen-proline, 2-oxoglutarate 4-dioxygenase (proline 4-hydroxylase), beta polypeptide	-4.233	$5.38x10^{-03}$
1554691_a_at	PACSIN2	protein kinase C and casein kinase substrate in neurons 2	-2.522	$4.83x10^{-04}$
211547_s_at	PAFAH1B1	platelet-activating factor acetylhydrolase, isoform Ib, alpha subunit 45kDa	-3.278	$2.27x10^{-03}$
200906_s_at	PALLD	palladin, cytoskeletal associated protein	-2.383	$1.28x10^{-03}$
200907_s_at	PALLD	palladin, cytoskeletal associated protein	-2.391	$1.62x10^{-03}$
222725_s_at	PALMD	palmdelphin	-3.222	$3.23x10^{-03}$
218947_s_at	PAPD1	PAP associated domain containing 1	-1.893	$8.86x10^{-03}$
226843_s_at	PAPD5	PAP associated domain containing 5	-1.907	$1.98x10^{-03}$
212720_at	PAPOLA	poly(A) polymerase alpha	-2.035	$3.64x10^{-03}$
210094_s_at	PARD3	par-3 partitioning defective 3 homolog (C. elegans)	-2.248	$6.93x10^{-03}$
221526_x_at	PARD3	par-3 partitioning defective 3 homolog (C. elegans)	-1.768	$1.98x10^{-03}$
1555175_a_at	PBLD	phenazine biosynthesis-like protein domain containing	-2.465	$2.35x10^{-04}$
224152_s_at	PBRM1	polybromo 1	-3.719	$2.27x10^{-03}$
209997_x_at	PCM1	pericentriolar material 1	-2.113	$7.32x10^{-04}$
203803_at	PCYOX1	prenylcysteine oxidase 1	-2.421	$9.81x10^{-04}$
224046_s_at	PDE7A	phosphodiesterase 7A	-2.650	$1.15x10^{-03}$

Additional File 2. Significant Genes in the Small Airways Epithelium of Smokers with COPD Between Chips that Failed QC and Chips that Passed QC[1] (cont., page 18)

Probe set ID	Gene symbol	Gene title	Fold-change (Fail QC/Pass QC)	p value
1552931_a_at	PDE8A	phosphodiesterase 8A	-2.076	$6.30x10^{-04}$
203242_s_at	PDLIM5	PDZ and LIM domain 5	-3.917	$9.69x10^{-03}$
211681_s_at	PDLIM5	PDZ and LIM domain 5	-3.476	$1.86x10^{-03}$
216804_s_at	PDLIM5	PDZ and LIM domain 5	-2.627	$9.23x10^{-04}$
210041_s_at	PGM3	phosphoglucomutase 3	-2.349	$5.01x10^{-03}$
201120_s_at	PGRMC1	progesterone receptor membrane component 1	-4.394	$1.62x10^{-03}$
1554153_a_at	PHF21A	PHD finger protein 21A	-2.463	$5.00x10^{-04}$
215236_s_at	PICALM	phosphatidylinositol binding clathrin assembly protein	-4.278	$4.82x10^{-05}$
215832_x_at	PICALM	phosphatidylinositol binding clathrin assembly protein	-2.055	$3.72x10^{-03}$
1563111_a_at	PIGX	phosphatidylinositol glycan anchor biosynthesis, class X	-1.986	$1.62x10^{-03}$
224661_at	PIGY	phosphatidylinositol glycan anchor biosynthesis, class Y	-2.237	$3.95x10^{-03}$
1553694_a_at	PIK3C2A	phosphoinositide-3-kinase, class 2, alpha polypeptide	-2.998	$3.07x10^{-04}$
201927_s_at	PKP4	plakophilin 4	-2.479	$2.93x10^{-03}$
203896_s_at	PLCB4	phospholipase C, beta 4	-2.047	$2.27x10^{-03}$
216633_s_at	PLCH1	phospholipase C, eta 1	-4.271	$2.16x10^{-03}$
205203_at	PLD1	phospholipase D1, phosphatidylcholine-specific	-1.896	$1.08x10^{-03}$
215807_s_at	PLXNB1	plexin B1	-3.273	$4.16x10^{-03}$
214526_x_at	PMS2L1	postmeiotic segregation increased 2-like 1 pseudogene	-1.674	$9.96x10^{-03}$
214756_x_at	PMS2L1	postmeiotic segregation increased 2-like 1 pseudogene	-1.713	$4.70x10^{-03}$
216843_x_at	PMS2L1	postmeiotic segregation increased 2-like 1 pseudogene	-1.841	$9.05x10^{-03}$
210707_x_at	PMS2L11	postmeiotic segregation increased 2-like 11 pseudogene	-2.022	$7.88x10^{-04}$
215412_x_at	PMS2L2	postmeiotic segregation increased 2-like 2 pseudogene	-1.911	$3.70x10^{-03}$
214473_x_at	PMS2L3	postmeiotic segregation increased 2-like 3	-2.175	$1.31x10^{-04}$
216111_x_at	PMS2L3	postmeiotic segregation increased 2-like 3	-1.695	$1.31x10^{-04}$
216525_x_at	PMS2L3	postmeiotic segregation increased 2-like 3	-2.200	$1.20x10^{-03}$
223309_x_at	PNPLA8	patatin-like phospholipase domain containing 8	-2.543	$4.82x10^{-05}$
1555383_a_at	POF1B	premature ovarian failure, 1B	-4.175	$7.38x10^{-03}$
215357_s_at	POLDIP3	polymerase (DNA-directed), delta interacting protein 3	-1.914	$6.73x10^{-03}$
207515_s_at	POLR1C	polymerase (RNA) I polypeptide C, 30kDa	-2.001	$1.81x10^{-03}$
212178_s_at	POM121 /// POM121C	POM121 membrane glycoprotein (rat) /// POM121 membrane glycoprotein C	-2.032	$9.24x10^{-03}$

Additional File 2. Significant Genes in the Small Airways Epithelium of Smokers with COPD Between Chips that Failed QC and Chips that Passed QC[1] (cont., page 19)

Probe set ID	Gene symbol	Gene title	Fold-change (Fail QC/Pass QC)	p value
208994_s_at	PPIG	peptidylprolyl isomerase G (cyclophilin G)	-2.664	$9.88x10^{-03}$
201702_s_at	PPP1R10	protein phosphatase 1, regulatory (inhibitor) subunit 10	-10.228	$1.46x10^{-03}$
203056_s_at	PRDM2	PR domain containing 2, with ZNF domain	-3.462	$3.78x10^{-03}$
201835_s_at	PRKAB1	protein kinase, AMP-activated, beta 1 non-catalytic subunit	-2.748	$9.24x10^{-03}$
1558027_s_at	PRKAB2	protein kinase, AMP-activated, beta 2 non-catalytic subunit	-2.202	$1.63x10^{-03}$
202742_s_at	PRKACB	protein kinase, cAMP-dependent, catalytic, beta	-2.941	$7.36x10^{-05}$
222615_s_at	PRKRIP1	PRKR interacting protein 1 (IL11 inducible)	-1.553	$2.97x10^{-03}$
204060_s_at	PRKX /// PRKY	protein kinase, X-linked /// protein kinase, Y-linked	-2.090	$3.62x10^{-03}$
206445_s_at	PRMT1	protein arginine methyltransferase 1	-2.170	$4.51x10^{-03}$
215707_s_at	PRNP	prion protein	-2.303	$9.83x10^{-04}$
213729_at	PRPF40A	PRP40 pre-mRNA processing factor 40 homolog A (S. cerevisiae)	-2.289	$3.82x10^{-03}$
214941_s_at	PRPF40A	PRP40 pre-mRNA processing factor 40 homolog A (S. cerevisiae)	-2.275	$3.20x10^{-04}$
211090_s_at	PRPF4B	PRP4 pre-mRNA processing factor 4 homolog B (yeast)	-4.537	$6.47x10^{-04}$
208879_x_at	PRPF6	PRP6 pre-mRNA processing factor 6 homolog (S. cerevisiae)	-3.356	$1.19x10^{-03}$
208447_s_at	PRPS1	phosphoribosyl pyrophosphate synthetase 1	-2.607	$2.60x10^{-03}$
232215_x_at	PRR11	proline rich 11	2.129	$7.46x10^{-03}$
221734_at	PRRC1	proline-rich coiled-coil 1	-2.643	$6.19x10^{-03}$
205515_at	PRSS12	protease, serine, 12 (neurotrypsin, motopsin)	-3.089	$4.64x10^{-03}$
207782_s_at	PSEN1	presenilin 1	-2.665	$2.82x10^{-03}$
201198_s_at	PSMD1	proteasome (prosome, macropain) 26S subunit, non-ATPase, 1	-2.532	$4.09x10^{-03}$
1554577_a_at	PSMD10	proteasome (prosome, macropain) 26S subunit, non-ATPase, 10	-2.264	$2.93x10^{-03}$
201388_at	PSMD3	proteasome (prosome, macropain) 26S subunit, non-ATPase, 3	-3.000	$2.99x10^{-03}$
200987_x_at	PSME3	proteasome (prosome, macropain) activator subunit 3 (PA28 gamma; Ki)	-2.070	$7.50x10^{-03}$
212220_at	PSME4	proteasome (prosome, macropain) activator subunit 4	-2.348	$6.97x10^{-03}$
222611_s_at	PSPC1	paraspeckle component 1	-2.453	$1.62x10^{-03}$
1555097_a_at	PTGFR	prostaglandin F receptor (FP)	-3.397	$3.92x10^{-04}$
217777_s_at	PTPLAD1	protein tyrosine phosphatase-like A domain containing 1	-3.323	$6.00x10^{-03}$
209895_at	PTPN11	protein tyrosine phosphatase, non-receptor type 11	-3.506	$4.51x10^{-03}$

Additional File 2. Significant Genes in the Small Airways Epithelium of Smokers with COPD Between Chips that Failed QC and Chips that Passed QC[1] (cont., page 20)

Probe set ID	Gene symbol	Gene title	Fold-change (Fail QC/Pass QC)	p value
209896_s_at	PTPN11	protein tyrosine phosphatase, non-receptor type 11	-2.864	$5.96x10^{-04}$
216915_s_at	PTPN12	protein tyrosine phosphatase, non-receptor type 12	-5.075	$4.34x10^{-03}$
40524_at	PTPN21	protein tyrosine phosphatase, non-receptor type 21	-1.675	$8.77x10^{-03}$
213799_s_at	PTPRA	protein tyrosine phosphatase, receptor type, A	-6.143	$7.63x10^{-03}$
212587_s_at	PTPRC	protein tyrosine phosphatase, receptor type, C	-4.478	$9.04x10^{-03}$
200635_s_at	PTPRF	protein tyrosine phosphatase, receptor type, F	-2.226	$3.63x10^{-03}$
211534_x_at	PTPRN2	protein tyrosine phosphatase, receptor type, N polypeptide 2	-2.286	$1.35x10^{-03}$
229982_at	QSER1	glutamine and serine rich 1	-3.576	$2.81x10^{-03}$
231830_x_at	RAB11FIP1	RAB11 family interacting protein 1 (class I)	-2.295	$1.05x10^{-03}$
207018_s_at	RAB27B	RAB27B, member RAS oncogene family	-2.008	$5.33x10^{-03}$
208734_x_at	RAB2A	RAB2A, member RAS oncogene family	-1.663	$9.32x10^{-03}$
206113_s_at	RAB5A	RAB5A, member RAS oncogene family	-2.957	$1.67x10^{-03}$
201048_x_at	RAB6A	RAB6A, member RAS oncogene family	-5.857	$4.28x10^{-03}$
220500_s_at	RABL2A /// RABL2B	RAB, member of RAS oncogene family-like 2A /// RAB, member of RAS oncogene family-like 2B	-3.095	$3.19x10^{-03}$
226090_x_at	RABL3	RAB, member of RAS oncogene family-like 3	-2.037	$2.03x10^{-03}$
210826_x_at	RAD17	RAD17 homolog (S. pombe)	-1.672	$1.03x10^{-03}$
211228_s_at	RAD17	RAD17 homolog (S. pombe)	-1.687	$1.86x10^{-03}$
200607_s_at	RAD21	RAD21 homolog (S. pombe)	-2.387	$5.00x10^{-04}$
208393_s_at	RAD50	RAD50 homolog (S. cerevisiae)	-1.898	$9.51x10^{-03}$
202101_s_at	RALB	v-ral simian leukemia viral oncogene homolog B (ras related; GTP binding protein)	-2.335	$9.90x10^{-03}$
209051_s_at	RALGDS	ral guanine nucleotide dissociation stimulator	-2.863	$6.47x10^{-04}$
201711_x_at	RANBP2	RAN binding protein 2	-2.675	$2.72x10^{-03}$
202583_s_at	RANBP9	RAN binding protein 9	-2.515	$8.02x10^{-03}$
216125_s_at	RANBP9	RAN binding protein 9	-2.059	$4.55x10^{-03}$
206220_s_at	RASA3	RAS p21 protein activator 3	-6.462	$2.73x10^{-03}$
211540_s_at	RB1	retinoblastoma 1	-3.184	$5.84x10^{-03}$
221440_s_at	RBBP9	retinoblastoma binding protein 9	-3.916	$9.43x10^{-03}$
232751_at	RBBP9	retinoblastoma binding protein 9	-4.510	$2.04x10^{-03}$
212332_at	RBL2	retinoblastoma-like 2 (p130)	-3.593	$3.55x10^{-03}$
222527_s_at	RBM22	RNA binding motif protein 22	-3.213	$4.82x10^{-05}$
212028_at	RBM25	RNA binding motif protein 25	-2.836	$4.17x10^{-04}$
229440_at	RBM47	RNA binding motif protein 47	-8.291	$1.48x10^{-04}$
201394_s_at	RBM5	RNA binding motif protein 5	-2.542	$8.96x10^{-03}$
209936_at	RBM5	RNA binding motif protein 5	-3.470	$1.81x10^{-03}$
213901_x_at	RBM9	RNA binding motif protein 9	-2.564	$3.93x10^{-03}$
220202_s_at	RC3H2	ring finger and CCCH-type zinc finger domains 2	-2.109	$8.14x10^{-03}$

Additional File 2. Significant Genes in the Small Airways Epithelium of Smokers with COPD Between Chips that Failed QC and Chips that Passed QC[1] (cont., page 21)

Probe set ID	Gene symbol	Gene title	Fold-change (Fail QC/Pass QC)	p value
218344_s_at	RCOR3	REST corepressor 3	-1.649	$7.82x10^{-03}$
34063_at	RECQL5	RecQ protein-like 5	-2.633	$4.13x10^{-03}$
222501_s_at	REPIN1	replication initiator 1	-3.483	$7.09x10^{-03}$
1566472_s_at	RETSAT	retinol saturase (all-trans-retinol 13,14-reductase)	-3.336	$1.56x10^{-03}$
207936_x_at	RFPL3	ret finger protein-like 3	1.701	$1.88x10^{-03}$
208031_s_at	RFX2	regulatory factor X, 2 (influences HLA class II expression)	-4.228	$3.62x10^{-03}$
238810_at	RFX3	regulatory factor X, 3 (influences HLA class II expression)	-2.431	$3.15x10^{-03}$
210676_x_at	RGPD5 /// RGPD6 /// RGPD8	RANBP2-like and GRIP domain containing 5 /// RANBP2-like and GRIP domain containing 6 /// RANBP2-like and GRIP domain containing 8	-1.712	$5.01x10^{-03}$
232053_x_at	RHBDD2	rhomboid domain containing 2	-2.559	$5.00x10^{-03}$
1554897_s_at	RHBDL2	rhomboid, veinlet-like 2 (Drosophila)	-2.244	$5.76x10^{-03}$
1570253_a_at	RHEBL1	Ras homolog enriched in brain like 1	-2.243	$6.80x10^{-03}$
1553962_s_at	RHOB	ras homolog gene family, member B	-16.811	$2.55x10^{-06}$
202129_s_at	RIOK3	RIO kinase 3 (yeast)	-2.748	$2.00x10^{-03}$
215588_x_at	RIOK3	RIO kinase 3 (yeast)	1.770	$1.52x10^{-03}$
209941_at	RIPK1	receptor (TNFRSF)-interacting serine-threonine kinase 1	-3.230	$3.78x10^{-03}$
234730_s_at	RIPK4	receptor-interacting serine-threonine kinase 4	-8.320	$1.42x10^{-03}$
211515_s_at	RIPK5	receptor interacting protein kinase 5	-1.984	$2.55x10^{-03}$
201823_s_at	RNF14	ring finger protein 14	-1.906	$8.21x10^{-03}$
1554312_at	RNF170	ring finger protein 170	1.545	$7.87x10^{-03}$
210932_s_at	RNF6	ring finger protein (C3H2C3 type) 6	-5.176	$2.23x10^{-03}$
221194_s_at	RNFT1	ring finger protein, transmembrane 1	-2.585	$9.14x10^{-04}$
204207_s_at	RNGTT	RNA guanylyltransferase and 5'-phosphatase	-1.725	$8.93x10^{-03}$
207223_s_at	ROD1	ROD1 regulator of differentiation 1 (S. pombe)	-2.370	$2.77x10^{-03}$
214697_s_at	ROD1	ROD1 regulator of differentiation 1 (S. pombe)	-2.049	$5.01x10^{-03}$
224826_at	RP5-1022P6.2	hypothetical protein KIAA1434	-1.604	$5.90x10^{-03}$
1557984_s_at	RPAP3	RNA polymerase II associated protein 3	-2.695	$2.22x10^{-03}$
212044_s_at	RPL27A	Ribosomal protein L27a	2.654	$4.33x10^{-03}$
214041_x_at	RPL37A	Ribosomal protein L37a	2.355	$8.71x10^{-03}$
221943_x_at	RPL38	Ribosomal protein L38	2.715	$5.33x10^{-03}$
222559_s_at	RPRD1A	regulation of nuclear pre-mRNA domain containing 1A	-2.434	$7.66x10^{-03}$
213350_at	RPS11	Ribosomal protein S11	3.306	$4.46x10^{-03}$
1555916_at	RPUSD3	RNA pseudouridylate synthase domain containing 3	1.523	$1.57x10^{-03}$
201203_s_at	RRBP1	ribosome binding protein 1 homolog 180kDa	-6.417	$1.63x10^{-03}$

Additional File 2. Significant Genes in the Small Airways Epithelium of Smokers with COPD Between Chips that Failed QC and Chips that Passed QC[1] (cont., page 22)

Probe set ID	Gene symbol	Gene title	Fold-change (Fail QC/Pass QC)	p value
		(dog)		
201206_s_at	RRBP1	ribosome binding protein 1 homolog 180kDa (dog)	-2.763	$1.31x10^{-04}$
201476_s_at	RRM1	ribonucleotide reductase M1	-1.943	$9.24x10^{-03}$
202301_s_at	RSRC2	arginine/serine-rich coiled-coil 2	-2.136	$5.14x10^{-03}$
210251_s_at	RUFY3	RUN and FYVE domain containing 3	-1.980	$7.46x10^{-03}$
215099_s_at	RXRB	retinoid X receptor, beta	-9.426	$7.50x10^{-03}$
1555618_s_at	SAE1	SUMO1 activating enzyme subunit 1	-2.416	$7.94x10^{-03}$
1559883_s_at	SAMHD1	SAM domain and HD domain 1	-3.176	$1.52x10^{-03}$
1570210_x_at	SAPS2	SAPS domain family, member 2	1.931	$1.06x10^{-03}$
209127_s_at	SART3	squamous cell carcinoma antigen recognized by T cells 3	-6.573	$5.68x10^{-03}$
222573_s_at	SAV1	salvador homolog 1 (Drosophila)	-2.651	$1.86x10^{-03}$
1554089_s_at	SBDS /// SBDSP	Shwachman-Bodian-Diamond syndrome /// Shwachman-Bodian-Diamond syndrome pseudogene	-2.902	$5.57x10^{-03}$
1552978_a_at	SCAMP1	secretory carrier membrane protein 1	-4.259	$1.48x10^{-04}$
206667_s_at	SCAMP1	secretory carrier membrane protein 1	-5.415	$8.86x10^{-03}$
206668_s_at	SCAMP1	secretory carrier membrane protein 1	-2.944	$4.79x10^{-04}$
201646_at	SCARB2	scavenger receptor class B, member 2	-2.475	$1.37x10^{-03}$
201647_s_at	SCARB2	scavenger receptor class B, member 2	-2.275	$6.60x10^{-03}$
202082_s_at	SEC14L1	SEC14-like 1 (S. cerevisiae)	-1.992	$5.27x10^{-03}$
216392_s_at	SEC23IP	SEC23 interacting protein	-3.119	$5.57x10^{-04}$
222385_x_at	SEC61A1	Sec61 alpha 1 subunit (S. cerevisiae)	-5.188	$2.61x10^{-04}$
201914_s_at	SEC63	SEC63 homolog (S. cerevisiae)	-2.603	$7.25x10^{-03}$
203788_s_at	SEMA3C	sema domain, immunoglobulin domain (Ig), short basic domain, secreted, (semaphorin) 3C	-2.332	$1.01x10^{-03}$
1552812_a_at	SENP1	SUMO1/sentrin specific peptidase 1	-3.413	$7.50x10^{-03}$
214720_x_at	SEPT10	septin 10	-2.104	$1.38x10^{-03}$
1554747_a_at	SEPT2	septin 2	-3.269	$2.68x10^{-04}$
200778_s_at	SEPT2	septin 2	-2.817	$6.02x10^{-03}$
209669_s_at	SERBP1	SERPINE1 mRNA binding protein 1	-2.443	$8.98x10^{-03}$
201965_s_at	SETX	senataxin	-1.981	$9.96x10^{-03}$
201070_x_at	SF3B1	splicing factor 3b, subunit 1, 155kDa	-2.764	$5.29x10^{-04}$
214305_s_at	SF3B1	splicing factor 3b, subunit 1, 155kDa	-2.564	$7.38x10^{-04}$
201742_x_at	SFRS1	splicing factor, arginine/serine-rich 1	-3.328	$1.86x10^{-03}$
225507_at	SFRS18	splicing factor, arginine/serine-rich 18	-2.439	$4.70x10^{-03}$
209376_x_at	SFRS2IP	splicing factor, arginine/serine-rich 2, interacting protein	-4.134	$6.33x10^{-03}$
213936_x_at	SFTPB	surfactant protein B	2.066	$9.21x10^{-03}$
205367_at	SH2B2	SH2B adaptor protein 2	1.587	$1.48x10^{-03}$
1558647_at	SH3D19	SH3 domain containing 19	-4.231	$9.64x10^{-04}$
224853_at	SLAIN2	SLAIN motif family, member 2	-2.431	$3.98x10^{-03}$
233230_s_at	SLAIN2	SLAIN motif family, member 2	-2.721	$1.81x10^{-03}$

Additional File 2. Significant Genes in the Small Airways Epithelium of Smokers with COPD Between Chips that Failed QC and Chips that Passed QC[1] (cont., page 23)

Probe set ID	Gene symbol	Gene title	Fold-change (Fail QC/Pass QC)	p value
203123_s_at	SLC11A2	solute carrier family 11 (proton-coupled divalent metal ion transporters), member 2	-2.085	$2.88x10^{-03}$
203125_x_at	SLC11A2	solute carrier family 11 (proton-coupled divalent metal ion transporters), member 2	-3.537	$2.35x10^{-04}$
244353_s_at	SLC2A12	solute carrier family 2 (facilitated glucose transporter), member 12	-1.612	$5.57x10^{-04}$
220796_x_at	SLC35E1	solute carrier family 35, member E1	2.567	$2.18x10^{-03}$
1556551_s_at	SLC39A6	solute carrier family 39 (zinc transporter), member 6	-4.430	$5.15x10^{-03}$
202089_s_at	SLC39A6	solute carrier family 39 (zinc transporter), member 6	-2.372	$1.97x10^{-03}$
219869_s_at	SLC39A8	solute carrier family 39 (zinc transporter), member 8	-2.281	$8.69x10^{-03}$
1555203_s_at	SLC44A4	solute carrier family 44, member 4	-5.195	$1.56x10^{-04}$
210738_s_at	SLC4A4	solute carrier family 4, sodium bicarbonate cotransporter, member 4	-2.961	$3.65x10^{-03}$
1554113_a_at	SLC4A8	solute carrier family 4, sodium bicarbonate cotransporter, member 8	-2.189	$1.41x10^{-03}$
205920_at	SLC6A6	solute carrier family 6 (neurotransmitter transporter, taurine), member 6	-8.750	$3.78x10^{-03}$
207626_s_at	SLC7A2	solute carrier family 7 (cationic amino acid transporter, y+ system), member 2	-4.419	$9.79x10^{-03}$
203579_s_at	SLC7A6	solute carrier family 7 (cationic amino acid transporter, y+ system), member 6	-1.979	$1.78x10^{-03}$
1553055_a_at	SLFN5	schlafen family member 5	-4.184	$6.93x10^{-04}$
1557078_at	SLFN5	schlafen family member 5	-8.717	$2.80x10^{-04}$
243999_at	SLFN5	schlafen family member 5	-4.287	$3.93x10^{-03}$
212257_s_at	SMARCA2	SWI/SNF related, matrix associated, actin dependent regulator of chromatin, subfamily a, member 2	-3.157	$4.51x10^{-04}$
201073_s_at	SMARCC1	SWI/SNF related, matrix associated, actin dependent regulator of chromatin, subfamily c, member 1	-2.788	$2.23x10^{-03}$
209257_s_at	SMC3	structural maintenance of chromosomes 3	-7.260	$4.82x10^{-05}$
212577_at	SMCHD1	structural maintenance of chromosomes flexible hinge domain containing 1	-2.402	$2.89x10^{-03}$
238434_at	SMCR8	Smith-Magenis syndrome chromosome region, candidate 8	-2.356	$4.03x10^{-03}$
224474_x_at	SMEK2	SMEK homolog 2, suppressor of mek1 (Dictyostelium)	-2.867	$9.23x10^{-04}$
205329_s_at	SNX4	sorting nexin 4	-1.754	$9.45x10^{-03}$
223028_s_at	SNX9	sorting nexin 9	-2.002	$3.51x10^{-04}$
206748_s_at	SPAG9	sperm associated antigen 9	-4.621	$2.73x10^{-03}$
230723_at	SPATA18	spermatogenesis associated 18 homolog (rat)	-4.380	$6.47x10^{-04}$

Additional File 2. Significant Genes in the Small Airways Epithelium of Smokers with COPD Between Chips that Failed QC and Chips that Passed QC[1] (cont., page 24)

Probe set ID	Gene symbol	Gene title	Fold-change (Fail QC/Pass QC)	p value
220298_s_at	SPATA6	spermatogenesis associated 6	-2.610	$2.60x10^{-04}$
220299_at	SPATA6	spermatogenesis associated 6	-2.035	$9.80x10^{-03}$
215383_x_at	SPG21	spastic paraplegia 21 (autosomal recessive, Mast syndrome)	1.789	$5.07x10^{-03}$
230884_s_at	SPG7	spastic paraplegia 7 (pure and complicated autosomal recessive)	-2.668	$2.65x10^{-03}$
225018_at	SPIRE1	spire homolog 1 (Drosophila)	-1.999	$5.27x10^{-03}$
210693_at	SPPL2B	signal peptide peptidase-like 2B	-10.069	$3.94x10^{-03}$
200671_s_at	SPTBN1	spectrin, beta, non-erythrocytic 1	-7.492	$1.88x10^{-03}$
213562_s_at	SQLE	squalene epoxidase	-2.779	$9.23x10^{-03}$
212058_at	SR140	U2-associated SR140 protein	-1.968	$9.50x10^{-03}$
209954_x_at	SS18	synovial sarcoma translocation, chromosome 18	-2.459	$2.39x10^{-03}$
216684_s_at	SS18	synovial sarcoma translocation, chromosome 18	-2.189	$6.92x10^{-03}$
201138_s_at	SSB	Sjogren syndrome antigen B (autoantigen La)	-2.493	$8.87x10^{-04}$
203015_s_at	SSX2IP	synovial sarcoma, X breakpoint 2 interacting protein	-1.961	$1.84x10^{-03}$
214971_s_at	ST6GAL1	ST6 beta-galactosamide alpha-2,6-sialyltranferase 1	-6.144	$6.04x10^{-03}$
210073_at	ST8SIA1	ST8 alpha-N-acetyl-neuraminide alpha-2,8-sialyltransferase 1	-1.753	$7.38x10^{-03}$
AFFX-HUMISGF3A/M97935_5_at	STAT1	signal transducer and activator of transcription 1, 91kDa	-3.828	$2.27x10^{-03}$
AFFX-HUMISGF3A/M97935_MA_at	STAT1	signal transducer and activator of transcription 1, 91kDa	-3.646	$2.49x10^{-03}$
AFFX-HUMISGF3A/M97935_MB_at	STAT1	signal transducer and activator of transcription 1, 91kDa	-2.773	$8.03x10^{-03}$
225246_at	STIM2	stromal interaction molecule 2	-1.837	$4.73x10^{-03}$
212009_s_at	STIP1	stress-induced-phosphoprotein 1	-3.818	$4.51x10^{-03}$
1553117_a_at	STK38	serine/threonine kinase 38	-3.493	$1.25x10^{-03}$
1558249_s_at	STX16	syntaxin 16	-2.568	$8.77x10^{-03}$
221638_s_at	STX16	syntaxin 16	-3.549	$1.63x10^{-03}$
233827_s_at	SUPT16H	suppressor of Ty 16 homolog (S. cerevisiae)	-10.234	$7.31x10^{-05}$
207540_s_at	SYK	spleen tyrosine kinase	-3.315	$7.70x10^{-03}$
209024_s_at	SYNCRIP	synaptotagmin binding, cytoplasmic RNA interacting protein	-3.315	$2.60x10^{-04}$
242774_at	SYNE2	spectrin repeat containing, nuclear envelope 2	-6.903	$1.41x10^{-03}$
1553033_at	SYTL5	synaptotagmin-like 5	-5.849	$2.48x10^{-03}$
201867_s_at	TBL1X	transducin (beta)-like 1X-linked	-3.177	$6.60x10^{-04}$
222634_s_at	TBL1XR1	transducin (beta)-like 1 X-linked receptor 1	-2.586	$6.08x10^{-03}$

Additional File 2. Significant Genes in the Small Airways Epithelium of Smokers with COPD Between Chips that Failed QC and Chips that Passed QC[1] (cont., page 25)

Probe set ID	Gene symbol	Gene title	Fold-change (Fail QC/Pass QC)	p value
212385_at	TCF4	transcription factor 4	-2.097	$5.00x10^{-03}$
1558702_at	TEX10	Testis expressed 10	-3.027	$1.10x10^{-03}$
207627_s_at	TFCP2	transcription factor CP2	-2.976	$2.50x10^{-04}$
237215_s_at	TFRC	transferrin receptor (p90, CD71)	-6.501	$2.61x10^{-03}$
203833_s_at	TGOLN2	trans-golgi network protein 2	-2.246	$5.03x10^{-04}$
212994_at	THOC2	THO complex 2	-2.739	$4.16x10^{-03}$
222439_s_at	THRAP3	thyroid hormone receptor associated protein 3	-13.618	$4.04x10^{-04}$
225730_s_at	THUMPD3	THUMP domain containing 3	-2.240	$2.19x10^{-03}$
1554890_a_at	TIA1	TIA1 cytotoxic granule-associated RNA binding protein	-2.610	$9.74x10^{-04}$
224676_at	TMED4	transmembrane emp24 protein transport domain containing 4	-2.231	$1.51x10^{-03}$
224321_at	TMEFF2	transmembrane protein with EGF-like and two follistatin-like domains 2	13.263	$2.44x10^{-03}$
226825_s_at	TMEM165	transmembrane protein 165	-2.639	$9.50x10^{-04}$
234726_s_at	TMEM168	transmembrane protein 168	-2.508	$4.17x10^{-03}$
225127_at	TMEM181	transmembrane protein 181	-1.750	$3.76x10^{-03}$
201001_s_at	TMEM189-UBE2V1 /// UBE2V1	TMEM189-UBE2V1 /// ubiquitin-conjugating enzyme E2 variant 1	-2.177	$6.92x10^{-03}$
232591_s_at	TMEM30A	transmembrane protein 30A	-3.596	$7.31x10^{-05}$
222418_s_at	TMEM43	transmembrane protein 43	-1.656	$2.60x10^{-04}$
204808_s_at	TMEM5	transmembrane protein 5	-2.345	$1.57x10^{-04}$
223948_s_at	TMPRSS3	transmembrane protease, serine 3	-2.730	$9.88x10^{-03}$
202643_s_at	TNFAIP3	tumor necrosis factor, alpha-induced protein 3	-2.107	$4.84x10^{-04}$
223827_at	TNFRSF19	tumor necrosis factor receptor superfamily, member 19	-4.187	$2.28x10^{-05}$
224090_s_at	TNFRSF19	tumor necrosis factor receptor superfamily, member 19	-5.597	$6.97x10^{-03}$
222563_s_at	TNKS2	tankyrase, TRF1-interacting ankyrin-related ADP-ribose polymerase 2	-3.072	$8.04x10^{-03}$
208900_s_at	TOP1	topoisomerase (DNA) I	-3.456	$4.76x10^{-03}$
201683_x_at	TOX4	TOX high mobility group box family member 4	-1.906	$5.90x10^{-04}$
201688_s_at	TPD52	tumor protein D52	-2.775	$1.37x10^{-03}$
214196_s_at	TPP1	tripeptidyl peptidase I	-2.335	$5.97x10^{-03}$
1569857_s_at	TPP2	tripeptidyl peptidase II	-3.485	$3.78x10^{-03}$
215220_s_at	TPR	translocated promoter region (to activated MET oncogene)	-6.438	$5.84x10^{-03}$
214327_x_at	TPT1	tumor protein, translationally-controlled 1	1.567	$5.27x10^{-03}$
201399_s_at	TRAM1	translocation associated membrane protein 1	-2.311	$5.51x10^{-03}$
214248_s_at	TRIM2	tripartite motif-containing 2	-2.343	$5.24x10^{-03}$
204911_s_at	TRIM3	tripartite motif-containing 3	-2.806	$1.84x10^{-03}$
203567_s_at	TRIM38	tripartite motif-containing 38	-2.061	$1.08x10^{-03}$

Additional File 2. Significant Genes in the Small Airways Epithelium of Smokers with COPD Between Chips that Failed QC and Chips that Passed QC[1] (cont., page 26)

Probe set ID	Gene symbol	Gene title	Fold-change (Fail QC/Pass QC)	p value
223131_s_at	TRIM8	tripartite motif-containing 8	-5.796	$4.09x10^{-03}$
225400_at	TSEN15	tRNA splicing endonuclease 15 homolog (S. cerevisiae)	-8.531	$1.72x10^{-04}$
218155_x_at	TSR1	TSR1, 20S rRNA accumulation, homolog (S. cerevisiae)	1.862	$3.86x10^{-03}$
233999_s_at	TTC26	tetratricopeptide repeat domain 26	-2.751	$1.66x10^{-03}$
208664_s_at	TTC3	tetratricopeptide repeat domain 3	-4.298	$1.10x10^{-06}$
226838_at	TTC32	tetratricopeptide repeat domain 32	-2.053	$9.14x10^{-04}$
202476_s_at	TUBGCP2	tubulin, gamma complex associated protein 2	-1.918	$1.81x10^{-03}$
212337_at	TUG1	taurine upregulated gene 1	-2.597	$8.41x10^{-04}$
209228_x_at	TUSC3	tumor suppressor candidate 3	-2.287	$9.72x10^{-03}$
214007_s_at	TWF1	twinfilin, actin-binding protein, homolog 1 (Drosophila)	-4.742	$1.48x10^{-04}$
219201_s_at	TWSG1	twisted gastrulation homolog 1 (Drosophila)	-2.537	$8.57x10^{-03}$
201008_s_at	TXNIP	thioredoxin interacting protein	-3.539	$5.73x10^{-04}$
201009_s_at	TXNIP	thioredoxin interacting protein	-2.120	$7.34x10^{-04}$
217799_x_at	UBE2H	ubiquitin-conjugating enzyme E2H (UBC8 homolog, yeast)	-2.550	$3.94x10^{-03}$
222420_s_at	UBE2H	ubiquitin-conjugating enzyme E2H (UBC8 homolog, yeast)	-2.592	$6.44x10^{-03}$
217826_s_at	UBE2J1	ubiquitin-conjugating enzyme E2, J1 (UBC6 homolog, yeast)	-2.234	$5.80x10^{-04}$
222395_s_at	UBE2Z	ubiquitin-conjugating enzyme E2Z	-1.794	$8.62x10^{-03}$
202316_x_at	UBE4B	ubiquitination factor E4B (UFD2 homolog, yeast)	-8.917	$3.71x10^{-03}$
238528_at	UBR1	ubiquitin protein ligase E3 component n-recognin 1	-2.028	$4.16x10^{-03}$
212007_at	UBXN4	UBX domain protein 4	-4.747	$1.40x10^{-03}$
212008_at	UBXN4	UBX domain protein 4	-3.480	$2.91x10^{-04}$
1554397_s_at	UEVLD	UEV and lactate/malate dehyrogenase domains	-3.852	$5.37x10^{-04}$
220958_at	ULK4	unc-51-like kinase 4 (C. elegans)	-2.494	$9.53x10^{-03}$
225869_s_at	UNC93B1	unc-93 homolog B1 (C. elegans)	-3.967	$2.61x10^{-03}$
201831_s_at	USO1	USO1 homolog, vesicle docking protein (yeast)	-4.830	$1.10x10^{-03}$
202412_s_at	USP1	ubiquitin specific peptidase 1	-3.506	$8.37x10^{-03}$
209136_s_at	USP10	ubiquitin specific peptidase 10	-2.805	$5.90x10^{-04}$
222616_s_at	USP16	ubiquitin specific peptidase 16	-3.379	$5.59x10^{-03}$
219211_at	USP18	ubiquitin specific peptidase 18	-2.098	$6.04x10^{-03}$
212065_s_at	USP34	ubiquitin specific peptidase 34	-2.457	$3.06x10^{-03}$
242647_at	USP34	ubiquitin specific peptidase 34	-2.587	$3.69x10^{-03}$
213023_at	UTRN	utrophin	-2.276	$2.60x10^{-04}$
203990_s_at	UTX	ubiquitously transcribed tetratricopeptide repeat, X chromosome	-2.481	$5.64x10^{-03}$

Additional File 2. Significant Genes in the Small Airways Epithelium of Smokers with COPD Between Chips that Failed QC and Chips that Passed QC[1] (cont., page 27)

Probe set ID	Gene symbol	Gene title	Fold-change (Fail QC/Pass QC)	p value
201337_s_at	VAMP3	vesicle-associated membrane protein 3 (cellubrevin)	-4.434	$5.30x10^{-03}$
224221_s_at	VAV3	vav 3 guanine nucleotide exchange factor	-5.893	$4.33x10^{-03}$
1553514_a_at	VNN3	vanin 3	-2.247	$9.82x10^{-03}$
222387_s_at	VPS35	vacuolar protein sorting 35 homolog (S. cerevisiae)	-4.291	$3.56x10^{-06}$
1555298_a_at	VWA3B	von Willebrand factor A domain containing 3B	-3.221	$2.27x10^{-03}$
1564598_a_at	VWA3B	von Willebrand factor A domain containing 3B	-3.250	$7.66x10^{-03}$
219679_s_at	WAC	WW domain containing adaptor with coiled-coil	-3.328	$9.90x10^{-03}$
210935_s_at	WDR1	WD repeat domain 1	-3.402	$6.35x10^{-03}$
1564238_a_at	WDR49	WD repeat domain 49	-3.519	$7.66x10^{-03}$
243087_at	WDR63	WD repeat domain 63	-1.878	$7.15x10^{-04}$
224730_at	WDR68	WD repeat domain 68	-2.012	$7.51x10^{-03}$
1554141_s_at	WDR78	WD repeat domain 78	-2.131	$8.57x10^{-03}$
220769_s_at	WDR78	WD repeat domain 78	-2.500	$3.23x10^{-03}$
244038_at	WDR89	WD repeat domain 89	-1.896	$1.67x10^{-03}$
204710_s_at	WIPI2	WD repeat domain, phosphoinositide interacting 2	-2.116	$7.37x10^{-03}$
202132_at	WWTR1	WW domain containing transcription regulator 1	-2.193	$2.61x10^{-03}$
208459_s_at	XPO7	exportin 7	-2.704	$3.71x10^{-03}$
233878_s_at	XRN2	5'-3' exoribonuclease 2	-2.642	$2.03x10^{-03}$
230557_at	XRRA1	X-ray radiation resistance associated 1	-4.639	$8.03x10^{-03}$
221423_s_at	YIPF5	Yip1 domain family, member 5	-2.515	$4.73x10^{-03}$
214659_x_at	YLPM1	YLP motif containing 1	-1.970	$1.63x10^{-03}$
1564053_a_at	YTHDF3	YTH domain family, member 3	-3.654	$1.69x10^{-04}$
217717_s_at	YWHAB	tyrosine 3-monooxygenase/tryptophan 5-monooxygenase activation protein, beta polypeptide	-2.031	$7.25x10^{-03}$
201593_s_at	ZC3H15	zinc finger CCCH-type containing 15	-2.594	$6.47x10^{-04}$
226897_s_at	ZC3H7A	zinc finger CCCH-type containing 7A	-3.349	$3.74x10^{-03}$
224868_at	ZDHHC5	zinc finger, DHHC-type containing 5	-2.474	$9.99x10^{-03}$
217741_s_at	ZFAND5	zinc finger, AN1-type domain 5	-1.820	$8.71x10^{-03}$
201856_s_at	ZFR	zinc finger RNA binding protein	-2.158	$2.13x10^{-03}$
1554159_a_at	ZMYND11	zinc finger, MYND domain containing 11	-5.709	$5.64x10^{-03}$
202136_at	ZMYND11	zinc finger, MYND domain containing 11	-1.618	$3.78x10^{-03}$
1554433_a_at	ZNF146	zinc finger protein 146	-2.546	$1.69x10^{-04}$
203248_at	ZNF24	zinc finger protein 24	-2.311	$4.51x10^{-03}$
241738_at	ZNF250	zinc finger protein 250	1.731	$1.16x10^{-03}$
1555192_at	ZNF277	zinc finger protein 277	1.989	$9.11x10^{-03}$
220055_at	ZNF287	zinc finger protein 287	-2.096	$3.06x10^{-03}$

Additional File 2. Significant Genes in the Small Airways Epithelium of Smokers with COPD Between Chips that Failed QC and Chips that Passed QC[1] (cont., page 28)

Probe set ID	Gene symbol	Gene title	Fold-change (Fail QC/Pass QC)	p value
1555337_a_at	ZNF317	zinc finger protein 317	-4.930	3.94×10^{-03}
1555166_a_at	ZNF396	zinc finger protein 396	-4.288	8.46×10^{-03}
235271_s_at	ZNF397 /// ZNF397OS	zinc finger protein 397 /// zinc finger protein 397 opposite strand	-5.302	8.33×10^{-03}
1554249_a_at	ZNF638	zinc finger protein 638	-3.033	3.62×10^{-03}
215570_s_at	ZNF780A /// ZNF780B	zinc finger protein 780A /// zinc finger protein 780B	-7.198	2.18×10^{-03}
226315_at	ZNF830	zinc finger protein 830	-1.814	1.86×10^{-03}
218349_s_at	ZWILCH	Zwilch, kinetochore associated, homolog (Drosophila)	-2.544	6.14×10^{-03}
1555014_x_at	---	---	2.436	8.13×10^{-04}
1556416_s_at	---	---	-3.942	7.01×10^{-03}
1559436_x_at	---	---	2.183	7.71×10^{-03}
1565567_at	---	---	2.282	4.84×10^{-03}
204552_at	---	---	-2.157	6.93×10^{-03}
208246_x_at	---	---	2.159	5.27×10^{-03}
213642_at	---	---	2.352	3.55×10^{-03}
214001_x_at	---	---	2.381	1.12×10^{-03}
214902_x_at	---	---	1.873	9.20×10^{-03}
215378_at	---	---	1.998	7.25×10^{-03}
215628_x_at	---	---	1.920	2.27×10^{-03}
216499_at	---	---	1.798	3.86×10^{-03}
217347_at	---	---	2.205	8.72×10^{-03}
217713_x_at	---	---	2.031	7.52×10^{-03}
221155_x_at	---	---	2.415	3.33×10^{-03}
222316_at	---	---	2.337	3.78×10^{-03}
224375_at	---	---	3.932	5.91×10^{-03}
228541_x_at	---	---	1.816	5.11×10^{-04}
232150_at	---	---	2.452	2.77×10^{-03}
232495_x_at	---	---	1.947	8.77×10^{-03}
233449_at	---	---	2.032	7.47×10^{-04}
233775_x_at	---	---	2.006	4.73×10^{-03}
234788_x_at	---	---	1.941	2.13×10^{-03}
235750_at	---	---	1.671	3.55×10^{-03}
236072_at	---	---	2.113	2.21×10^{-03}
237885_at	---	---	-2.666	8.93×10^{-03}
238172_at	---	---	1.628	3.77×10^{-03}
238888_at	---	---	-1.790	2.80×10^{-03}
241417_at	---	---	1.948	2.48×10^{-03}
242598_at	---	---	2.113	3.55×10^{-03}
243442_x_at	---	---	2.790	8.11×10^{-03}
AFFX-BioB-3_at	---	---	3.416	1.10×10^{-03}
AFFX-BioB-5_at	---	---	3.167	8.41×10^{-04}
AFFX-BioB-M_at	---	---	3.598	5.16×10^{-04}

Additional File 2. Significant Genes in the Small Airways Epithelium of Smokers with COPD Between Chips that Failed QC and Chips that Passed QC[1] (cont., page 29)

Probe set ID	Gene symbol	Gene title	Fold-change (Fail QC/Pass QC)	p value
AFFX-BioC-3_at	---	---	3.307	$1.69x10^{-04}$
AFFX-BioC-5_at	---	---	3.578	$2.60x10^{-04}$
AFFX-BioDn-3_at	---	---	2.672	$1.08x10^{-03}$
AFFX-BioDn-5_at	---	---	2.907	$1.26x10^{-03}$
AFFX-CreX-3_at	---	---	2.641	$4.88x10^{-04}$
AFFX-CreX-5_at	---	---	3.019	$2.66x10^{-04}$
AFFX-r2-Ec-bioB-3_at	---	---	3.314	$2.06x10^{-04}$
AFFX-r2-Ec-bioB-5_at	---	---	3.467	$2.60x10^{-04}$
AFFX-r2-Ec-bioB-M_at	---	---	3.510	$4.51x10^{-04}$
AFFX-r2-Ec-bioC-3_at	---	---	3.036	$5.80x10^{-04}$
AFFX-r2-Ec-bioC-5_at	---	---	3.235	$1.39x10^{-03}$
AFFX-r2-Ec-bioD-3_at	---	---	2.580	$1.98x10^{-03}$
AFFX-r2-Ec-bioD-5_at	---	---	2.636	$2.44x10^{-03}$
AFFX-r2-P1-cre-3_at	---	---	2.325	$2.00x10^{-03}$
AFFX-r2-P1-cre-5_at	---	---	2.419	$1.59x10^{-03}$

[1] Shown are the 888 probe sets that are differentially expressed (using criteria of a fold change greater than 1.5 and a p value, with Benjamini-Hochberg correction, less than 0.01, in n=11 pass QC samples and n=11 fail QC samples, all from the small airway epithelium of individuals with COPD.
